# New Amino Naphthoquinone Derivatives as Anti-*Trypanosoma cruzi* Agents Targeting Trypanothione Reductase

**DOI:** 10.3390/pharmaceutics14061121

**Published:** 2022-05-25

**Authors:** Christian Espinosa-Bustos, Mariana Ortiz Pérez, Alonzo Gonzalez-Gonzalez, Ana María Zarate, Gildardo Rivera, Javier A. Belmont-Díaz, Emma Saavedra, Mauricio A. Cuellar, Karina Vázquez, Cristian O. Salas

**Affiliations:** 1 Departamento de Farmacia, Facultad de Química y de Farmacia, Pontificia Universidad Católica de Chile, Vicuña Mackenna 4860, Santiago 7820436, Chile; ccespino@uc.cl; 2 Departamento de Parasitología, Facultad de Medicina Veterinaria y Zootecnia, Universidad Autónoma de Nuevo León, Francisco Villa 20, General Escobedo 66054, Mexico; mariana.ortiz92@live.com.mx; 3 Laboratorio de Biotecnología Farmacéutica, Centro de Biotecnología Genómica, Instituto Politécnico Nacional, Boulevard del Maestro s/n, Reynosa 88710, Mexico; al.gonzalez.gonzalez88@gmail.com (A.G.-G.); gildardors@hotmail.com (G.R.); 4 Departamento de Química Orgánica, Facultad de Química y de Farmacia, Pontificia Universidad Católica de Chile, Vicuña Mackenna 4860, Macul, Santiago 7820436, Chile; amzarate@uc.cl; 5 Departamento de Bioquímica, Instituto Nacional de Cardiología Ignacio Chávez, México City 14080, Mexico; belmont81@hotmail.com (J.A.B.-D.); emma_saavedra2002@yahoo.com (E.S.); 6 Centro de Investigación Farmacopea Chilena, Escuela de Química y Farmacia, Facultad de Farmacia, Universidad de Valparaíso, Av. Gran Bretaña 1093, Valparaíso 2340000, Chile; mauricio.cuellar@uv.cl

**Keywords:** *Trypanosoma cruzi*, amino naphthoquinones, trypomastigote, epimastigote, trypanothione reductase, docking studies

## Abstract

To develop novel chemotherapeutic alternatives for the treatment of Chagas disease, in this study, a set of new amino naphthoquinone derivatives were synthesised and evaluated in vitro on the epimastigote and trypomastigote forms of *Trypanosoma cruzi* strains (NINOA and INC-5) and on J774 murine macrophages. The design of the new naphthoquinone derivatives considered the incorporation of nitrogenous fragments with different substitution patterns present in compounds with activity on *T. cruzi,* and, thus, 19 compounds were synthesised in a simple manner. Compounds **2e** and **7j** showed the lowest IC_50_ values (0.43 µM against both strains for **2e** and 0.19 µM and 0.92 µM for **7j**). Likewise, **7j** was more potent than the reference drug, benznidazole, and was more selective on epimastigotes. To postulate a possible mechanism of action, molecular docking studies were performed on *T. cruzi* trypanothione reductase (*Tc*TR), specifically at a site in the dimer interface, which is a binding site for this type of naphthoquinone. Interestingly, **7j** was one of the compounds that showed the best interaction profile on the enzyme; therefore, **7j** was evaluated on TR, which behaved as a non-competitive inhibitor. Finally, **7j** was predicted to have a good pharmacokinetic profile for oral administration. Thus, the naphthoquinone nucleus should be considered in the search for new trypanocidal agents based on our hit **7j**.

## 1. Introduction

American trypanosomiasis (also known as Chagas disease) is one of the most important neglected diseases affecting Latin America and is caused by the protozoan parasite *Trypanosoma cruzi* (*T. cruzi*), which is transmitted to humans by the bite of a triatomine bug [1,2]. Owing to migratory processes, the disease can be found in non-endemic places such as the United States and Europe, affecting approximately 11 million people worldwide [3]. However, there are only two approved drugs used to treat the infection, benznidazole (Bzn) and nifurtimox, both of which are frequently associated with unpleasant side effects and low effectiveness [4,5]. Thus, there is a need to find new compounds with trypanocidal activity which are safer and more effective than the canonical drugs [6,7].

**Figure 1:**
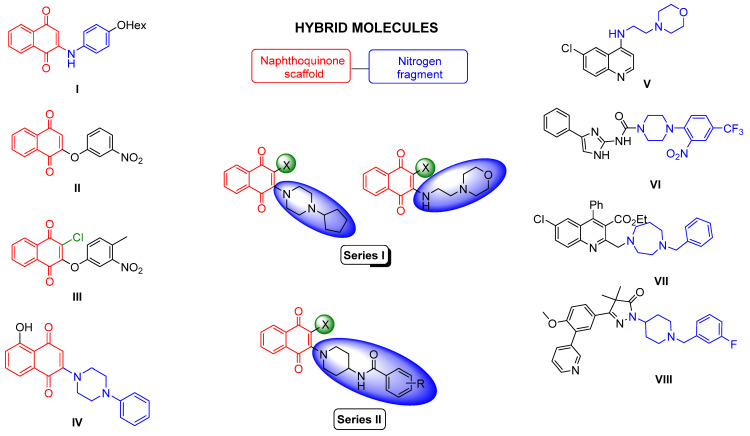
Chemical structures of trypanocidal quinone derivatives and design strategy for new anti-*T. cruzi* agents.

Compounds derived from natural or synthetic naphthoquinones have been considered as privileged scaffolds for obtaining novel agents with antiparasitic activity [8,9,10]. Our research group previously reported the synthesis and trypanocidal evaluation of a series of 2-anilino-1,4-naphthoquinones, highlighting compound **I** (Figure 1), which exhibited potent activity against epimastigotes of the Tulahuén strain (IC_50_ = 1.72 µM) and high selectivity against Vero cells [11]. Subsequently, we reported the synthesis and evaluation of a series of aryloxy-quinones against the Y strain of *T. cruzi* epimastigote, where 2-(3-nitrophenoxy)-1,4-naphthoquinone **II** (Figure 1) displayed remarkable nanomolar inhibitory activity (IC_50_ = 20 nM) and a high selectivity index (SI = 625) compared with J774 murine macrophages cells [12]. To evaluate our quinone derivatives on other *T. cruzi* strains as well as trypomastigote forms, we recently designed and synthetised a small series of aryloxy-naphthoquinones. Our best results were for compound **III**, which showed high potency on the NINOA strain of *T. cruzi* trypomastigotes (IC_50_ = 9.38 µM), low toxicity on J774 cells (IC_50_ = 117 µM), and was more potent and selective than Bzn (40.67 and 352 µM, respectively) [13].

It is well-known that some antiparasitic quinones also have potent antitumoral activity and share the same biological targets, likely due to their mechanisms of action [11,14]. One example of this behaviour was observed for compound **IV**, whose design was inspired by the antimalarial agent atovaquone and showed cytotoxic effects on several cancer cell lines and induced apoptosis in these cells [15].

In the search for new chemical entities to obtain anti-*T. cruzi* agents, the use of nitrogenous scaffolds has been well documented [16]. For example, Braga et al. [17], synthesised derivatives of quinolines, indoles, and pyrimidines substituted with amino-alkylmorpholines, which were shown to be active against this parasite. Compound **V** (Figure 1) exhibited an IC_50_ = 67.7 µM in trypomastigote cultures (Y strain). Another example of the use of nitrogenous heterocycles was presented by Araujo et al. [18], where the use of aryl-piperazines linked to the imidazole ring resulted in derivatives with activity on *T. cruzi* and *T. brucei*; derivative **VI** (Figure 1), in particular, showed high potency on *T. cruzi* trypomastigotes (IC_50_ = 2.2 µM), with a low toxicity on MCR-5 human lung fibroblasts. The use of arylpiperazine isosteres linked to different scaffolds has also provided compounds with activity on *T. cruzi*. Interesting examples include the 1-benzyl-1,4-diazepane fragment (**VII**, Figure 1), which exhibited powerful activity on epimastigotes, tripomastigotes and amastigotes [19], and the 1-benzylpiperidin-4-amine core (**VIII**, Figure 1), which showed activity against the intracellular amastigotes of *T. cruzi* (Tulahuén strain) [20].

Considering that the aforementioned compounds have provided chemical fragments to design novel anti-Chagasic agents, in this work, we synthesised and evaluated two series of hybrid molecules related to amino naphthoquinones against epimastigotes and trypomastigotes of two Mexican strains of *T. cruzi* (Figure 1). The design of **Series I** was based on some modifications of the naphthoquinone scaffold: (i) the incorporation of a nitrogen fragment at the C-2 or C-3 position, such as those present in compounds **V**, **VI**, and **VIII**, and (ii) the substitution of the hydrogen atom at the C-2 position by a halogen atom (chlorine or bromine), thanks to our previous observation that this modification enhanced selectivity against *T. cruzi* compared with mammalian cells [11,12]. For **Series II**, we decided to replace the nitrogenated fragment considered in **Series I** by a *N*-(piperidin-4-yl) benzamide moiety, with different substituents in the benzene core and the maintaining of the chlorine at the C-2 position. Subsequently, the biological effects on INC-5 and NINOA strains of all of the synthetised quinones were evaluated. In addition, to determine a possible mechanism of action for these quinone derivatives, molecular docking studies were performed on *T. cruzi* trypanothione reductase (*Tc*TR), as this protein plays a pivotal role in the redox metabolism of the parasite. This possible target has been previously studied by our group [21,22]. Finally, for the most promising trypanocidal quinone, we conducted an inhibition kinetics evaluation in terms of *Tc*TR.

## 2. Materials and Methods

### 2.1. General

The reagents and chemicals used in this work were obtained from Sigma Aldrich (San Louis, MO, USA). Those compounds that had already been published previously were synthesised according to the respective procedures, and this was indicated with the relevant references. The purity of all synthesised compounds was checked by NMR and TLC. In the case of TLC, silica gel 60 F_254_ 25 aluminium foils 20 × 20 C (purchased from Merck, Burlington, VT, USA) were used as a stationary phase and a mobile phase composed of petroleum ether and ethyl acetate in a ratio of 75:25. In the NMR spectra, the chemical shifts of each signal were reported in parts per million (ppm) and the coupling constants (J) in hertz (Hz). Also, for the multiplicity of the NMR signals, they are expressed as s (singlet), d (doublet), t (triplet), dd (doublet doublet), and bs (broad singlet), as appropriate.

### 2.2. Instrumentation

The following instruments were used for the identification and characterisation of each of the synthesised compounds: (1) Kofler Thermogerate apparatus (Reichert, Werke A.G., Wien, Austria) for the determination of the melting points (m.p.), which are expressed in degrees Celsius (°C) without corrections; (2) BRUKER AVANCE III HD-400 spectrometers (400 MHz (^1^H) and 100 MHz (^13^C)) or 200 MHz )200 MHz (^1^H) and 50 MHz (^13^C)) to obtain the ^1^H and ^13^C NMR spectra (tetramethylsilane, TMS, was used as an internal reference); (3) BRUKER COMPACT QTOF MS + Elute UHPLC with a constant nebuliser temperature of 250 °C, for the acquisition of HRMS-ESI data. For this, samples dissolved in acetonitrile were injected directly into the ESI source through an injection valve and by means of a syringe pump with a flow rate of 5 µL min^−1^. Measurements were carried out in the positive ion mode, with a scanning range of *m*/*z* 300.00–1510.40 and a resolution of 140,000.

### 2.3. Synthesis

#### 2.3.1. General Procedure for the Synthesis for Compounds **2a**–**i**

In a reaction flask, the suitable amine, halo-1,4-naphthoquinone (1.0 mmol), and DIEA (2.0 mmol) were suspended in acetonitrile (10 mL). The mixture was stirred for 4 h at room temperature. Then, the solvent was removed under a vacuum, and the crude product was purified by column chromatography on silica gel, using methylene chloride/acetone (8:1) as a mobile phase.

*2-((2-Morpholinoethyl)amino)naphthalene-1,4-dione **2a**.* Orange solid, m.p. 124–126 °C, yield 46% [23].

*2-Chloro-3-((2-morpholinoethyl)amino)naphthalene-1,4-dione **2b**.* Orange solid, m.p. 113–115 °C, yield 86% [24].

*2-Bromo-3-((2-morpholinoethyl)amino)naphthalene-1,4-dione **2c**.* Orange solid, m.p. 119–120 °C, yield 74%. ^1^H NMR (400 MHz, CDCl_3_) δ 8.08 (d, *J* = 7.6 Hz, 1H), 7.95 (d, *J* = 7.6 Hz, 1H), 7.65 (t, *J* = 7.5 Hz, 1H), 7.56 (t, *J* = 7.5 Hz, 1H), 6.92 (s, 1H), 3.91 (dd, *J* = 11.2, 5.6 Hz, 2H), 3.72–3.69 (m, 4H), 2.62 (t, *J* = 5.9 Hz, 2H), 2.49 (d, *J* = 3.9 Hz, 4H). ^13^C NMR (101 MHz, CDCl_3_) δ 180.24, 176.17, 146.98, 134.68, 132.38, 132.29 (2C), 130.02, 126.90, 126.71, 67.03 (2C), 56.63, 52.92 (2C), 41.16. HRMS calcd. for (C_16_H_18_BrN_2_O_3_ [M + H]^+^): 365.0495. Found: 365.0497.

*2-(4-Cyclopentylpiperazin-1-yl)naphthalene-1,4-dione **2d**.* Orange solid, m.p. 129–130 °C, yield 67%. ^1^H NMR (400 MHz, CDCl_3_) δ 7.98 (d, *J* = 7.4 Hz, 1H), 7.94 (d, *J* = 7.5 Hz, 1H), 7.63 (t, *J* = 7.1 Hz, 1H), 7.58 (t, *J* = 7.2 Hz, 1H), 5.96 (s, 1H), 3.55–3.46 (m, 4H), 2.68–2.58 (m, 4H), 2.56–2.44 (m, 1H), 1.84 (d, *J* = 5.8 Hz, 2H), 1.72–1.61 (m, 2H), 1.58–1.47 (m, 2H), 1.44–1.35 (m, 2H). ^13^C NMR (101 MHz, CDCl_3_) δ 183.64, 183.09, 153.70, 133.83, 132.83, 132.42, 132.37, 126.65, 125.53, 111.55, 67.27, 51.81 (2C), 48.84 (2C), 30.35 (2C), 24.10 (2C). HRMS calcd. for (C_19_H_23_N_2_O_2_ [M + H]^+^): 311.1754. Found: 311.1755.

*2-Chloro-3-(4-cyclopentylpiperazin-1-yl)naphthalene-1,4-dione **2e**.* Orange solid, m.p. 125–126 °C, yield 71%. ^1^H NMR (400 MHz, CDCl_3_) δ 8.05 (d, *J* = 7.1 Hz, 1H), 7.95 (d, *J* = 7.2 Hz, 1H), 7.68–7.55 (m, 2H), 3.66–3.59 (m, 4H), 2.65 (s, 4H), 2.60–2.49 (m, 1H), 1.85 (d, *J* = 5.6 Hz, 2H), 1.74–1.60 (m, 2H), 1.59–1.34 (m, 4H). ^13^C NMR (101 MHz, CDCl_3_) δ 181.87, 178.00, 149.83, 134.05, 133.02, 131.64, 131.45, 126.85, 126.54, 122.58, 67.45, 52.99 (2C), 51.36 (2C), 30.28 (2C), 24.10 (2C). HRMS calcd. for (C_19_H_21_ClN_2_O_2_ [M + H]^+^): 345.1364. Found: 345.1367.

*2-Bromo-3-(4-cyclopentylpiperazin-1-yl)naphthalene-1,4-dione **2f**.* Orange solid, m.p. 121–122 °C, yield 80%. ^1^H NMR (400 MHz, CDCl_3_) δ 8.05 (d, *J* = 8.1 Hz, 1H), 7.95 (d, *J* = 7.0 Hz, 1H), 7.67–7.57 (m, 2H), 3.67–3.59 (m, 4H), 2.66 (s, 4H), 2.59–2.49 (m, 1H), 1.84 (d, *J* = 6.0 Hz, 2H), 1.74–1.61 (m, 2H), 1.59–1.34 (m, 4H). ^13^C NMR (101 MHz, CDCl_3_) δ 181.60, 178.11, 152.53, 134.01, 133.00, 131.42, 131.34, 126.93, 126.87, 116.38, 67.44, 52.94 (2C), 51.77 (2C), 30.31 (2C), 24.11 (2C). HRMS calcd. for (C_19_H_21_BrN_2_O_2_ [M + H]^+^): 389.0859. Found: 389.0857.

*2-(4-Morpholinopiperidin-1-yl)naphthalene-1,4-dione **2g**.* Orange solid, m.p. 151–153 °C, yield 18%. ^1^H NMR (400 MHz, CDCl_3_) δ 7.99 (dd, *J* = 7.6, 1.0 Hz, 1H), 7.94 (dd, *J* = 7.6, 1.1 Hz, 1H), 7.64 (td, *J* = 7.5, 1.4 Hz, 1H), 7.59 (td, *J* = 7.5, 1.4 Hz, 1H), 5.99 (s, 1H), 4.05 (d, *J* = 13.1 Hz, 2H), 3.75–3.65 (m, 4H), 3.03–2.87 (m, 2H), 2.58–2.50 (m, 4H), 2.50–2.39 (m, 1H), 1.93 (d, *J* = 12.4 Hz, 2H), 1.66 (ddd, *J* = 15.7, 12.5, 3.9 Hz, 2H). ^13^C NMR (101 MHz, CDCl_3_) δ 183.57, 183.19, 153.62, 133.84, 132.84, 132.46, 132.37, 126.64, 125.51, 111.22, 67.17 (2C), 61.49, 49.76 (2C), 48.47 (2C), 28.06 (2C). HRMS calcd. for (C_19_H_22_N_2_O_3_ [M + H]^+^): 327.1703. Found: 327.1704.

*2-Chloro-3-(4-morpholinopiperidin-1-yl)naphthalene-1,4-dione **2h**.* Red solid, m.p. 145–146 °C, yield 31%. ^1^H NMR (400 MHz, CDCl_3_) δ 8.04 (dd, *J* = 7.4, 1.2 Hz, 1H), 7.94 (dd, *J* = 7.4, 1.3 Hz, 1H), 7.67–7.56 (m, 2H), 3.84 (d, *J* = 13.4 Hz, 2H), 3.72–3.66 (m, 4H), 3.28–3.20 (m, 2H), 2.58–2.53 (m, 4H), 2.43 (tt, *J* = 11.1, 3.5 Hz, 1H), 1.92 (d, *J* = 11.9 Hz, 2H), 1.70 (qd, *J* = 12.1, 3.8 Hz, 2H). ^13^C NMR (101 MHz, CDCl_3_) δ 181.91, 177.99, 150.32, 134.02, 133.03, 131.63, 131.47, 126.83, 126.52, 123.10, 67.17 (2C), 61.55, 51.04 (2C), 49.75 (2C), 29.31 (2C). HRMS calcd. for (C_19_H_21_ClN_2_O_3_ [M + H]^+^): 361.1313. Found: 361.1312.

*2-Bromo-3-(4-morpholinopiperidin-1-yl)naphthalene-1,4-dione **2i**.* Red Solid, m.p. 148–149 °C, yield 29%. ^1^H NMR (400 MHz, CDCl_3_) δ 8.05 (d, *J* = 7.8 Hz, 1H), 7.94 (d, *J* = 6.9 Hz, 1H), 7.62 (p, *J* = 7.1 Hz, 2H), 3.84 (d, *J* = 13.3 Hz, 2H), 3.73–3.65 (m, 4H), 3.26 (t, *J* = 12.4 Hz, 2H), 2.60–2.52 (m, 4H), 2.43 (ddd, *J* = 11.2, 7.8, 3.8 Hz, 1H), 1.92 (d, *J* = 12.0 Hz, 2H), 1.72 (qd, *J* = 12.1, 3.6 Hz, 2H). ^13^C NMR (101 MHz, CDCl_3_) δ 181.66, 178.12, 153.06, 133.99, 133.02, 131.44, 131.35, 126.92, 126.87, 117.10, 67.23 (2C), 61.51, 51.46 (2C), 49.78 (2C), 29.29 (2C). HRMS calcd. for (C_19_H_21_BrN_2_O_3_ [M + H]^+^): 407.0790. Found: 407.0790.

#### 2.3.2. General Procedure for the Synthesis for Compounds **5a**–**j**

In a reaction flask, the respective acyl chlorides **4a**–**j** (1.5 mmol), *tert*-butyl-4-aminopiperidine-1-carboxylate (1.0 mmol), and triethylamine (3.0 mmol) were added using anhydrous THF as a solvent (10 mL). The reaction mixture was stirred at room temperature for 3 h. The solvent was then removed under reduced pressure and the reaction crude was purified in a silica gel chromatographic column using methylene chloride as a mobile phase [25].

*tert-Butyl-4-(2-bromobenzamido)piperidine-1-carboxylate **5a**.* White solid, m.p. 125–126 °C, yield 64%. ^1^H NMR (200 MHz, CDCl_3_) δ 7.56 (dd, *J* = 7.7, 1.2 Hz, 1H), 7.48 (dd, *J* = 7.5, 1.8 Hz, 1H), 7.40–7.19 (m, 2H), 6.05 (d, *J* = 7.7 Hz, 1H), 4.24–3.93 (m, 3H), 3.03–2.80 (m, 2H), 2.02 (dd, *J* = 12.7, 3.1 Hz, 2H), 1.56–1.26 (m, 11H). ^13^C NMR (50 MHz, CDCl_3_) δ 166.93, 154.65, 137.75, 133.27, 131.22, 129.49, 127.55, 119.18, 79.68, 47.37, 42.55 (2C), 31.75 (2C), 28.40 (3C).

*tert-Butyl-4-(4-nitrobenzamido)piperidine-1-carboxylate **5b**.* Pale yellow solid, m.p. 167–169 °C, yield 72%. ^1^H NMR (200 MHz, CDCl_3_) δ 8.26 (d, *J* = 8.7 Hz, 2H), 7.95 (d, *J* = 8.7 Hz, 2H), 6.41 (d, *J* = 7.7 Hz, 1H), 4.26–3.92 (m, 3H), 2.90 (t, *J* = 12.0 Hz, 2H), 2.03 (d, *J* = 10.5 Hz, 2H), 1.58–1.23 (m, 11H). ^13^C NMR (50 MHz, CDCl_3_) δ 164.79, 154.67, 149.57, 140.07, 128.20 (2C), 123.74 (2C), 79.86, 47.72, 42.72 (2C), 31.99 (2C), 28.40 (3C).

*tert-Butyl-4-(3-chlorobenzamido)piperidine-1-carboxylate **5c***. White solid, m.p. 168–170 °C, yield 51%. ^1^H NMR (400 MHz, CDCl_3_) δ 7.72 (d, *J* = 1.6 Hz, 1H), 7.62 (d, *J* = 7.7 Hz, 1H), 7.43 (d, *J* = 8.7 Hz, 1H), 7.32 (t, *J* = 7.9 Hz, 1H), 6.37 (d, *J* = 7.7 Hz, 1H), 4.15–3.96 (m, 3H), 2.85 (t, *J* = 11.8 Hz, 2H), 1.97 (dd, *J* = 12.2, 2.2 Hz, 2H), 1.48–1.33 (m, 11H). ^13^C NMR (101 MHz, CDCl_3_) δ 165.56, 154.70, 136.36, 134.66, 131.46, 129.84, 127.30, 125.13, 79.74, 47.45, 42.76 (2C), 32.01 (2C), 28.42 (3C).

*tert-Butyl-4-(4-(chloromethyl)benzamido)piperidine-1-carboxylate **5d**.* White solid, m.p. 163–165 °C, yield 57%. ^1^H NMR (200 MHz, CDCl_3_) δ 7.68 (d, *J* = 8.1 Hz, 2H), 7.35 (d, *J* = 8.1 Hz, 2H), 6.25 (d, *J* = 7.7 Hz, 1H), 4.52 (s, 2H), 4.17–3.92 (m, 3H), 2.80 (t, *J* = 12.0 Hz, 2H), 2.00–1.86 (m, 2H), 1.49–1.20 (m, 11H). ^13^C NMR (50 MHz, CDCl_3_) δ 166.28, 154.68, 140.78, 134.53, 128.66 (2C), 127.40 (2C), 79.69, 47.31, 45.36, 42.74 (2C), 32.05 (2C), 28.41 (3C).

*tert-Butyl-4-(3,5-dimethylbenzamido)piperidine-1-carboxylate **5e**.* White solid, m.p. 177–179 °C, yield 70%. ^1^H NMR (200 MHz, CDCl_3_) δ 7.27 (s, 2H), 7.05 (s, 1H), 5.98 (d, *J* = 7.7 Hz, 2H), 4.18–3.89 (m, 3H), 2.95–2.73 (m, 2H), 2.27 (s, 6H), 1.93 (dd, *J* = 12.4, 2.9 Hz, 2H), 1.47–1.22 (m, 11H). ^13^C NMR (50 MHz, CDCl_3_) δ 167.23, 154.72, 138.26 (2C), 134.56, 133.06, 124.61 (2C), 79.65, 47.11, 42.75 (2C), 32.14 (2C), 28.42 (3C), 21.19 (2C).

*tert-Butyl-4-(3,5-dinitrobenzamido)piperidine-1-carboxylate **5f**.* White solid, m.p. 216–217 °C, yield 68%. ^1^H NMR (200 MHz, CDCl_3_) δ 9.13 (t, *J* = 1.9 Hz, 1H), 9.02 (d, *J* = 1.9 Hz, 2H), 7.11 (d, *J* = 7.7 Hz, 1H), 4.17 (d, *J* = 14.1 Hz, 3H), 2.91 (t, *J* = 12.3 Hz, 2H), 2.05 (d, *J* = 10.5 Hz, 2H), 1.64–1.35 (m, 11H). ^13^C NMR (50 MHz, CDCl_3_) δ 162.08, 154.77, 148.59 (2C), 137.95, 127.41 (2C), 121.00, 80.05, 48.18, 42.82 (2C), 31.85 (2C), 28.39 (3C).

*tert-Butyl-4-(3-chloro-5-fluorobenzamido)piperidine-1-carboxylate **5g**.* White solid, m.p. 172–174 °C, yield 42%. ^1^H NMR (200 MHz, CDCl_3_) δ 7.53 (s, 1H), 7.45–7.35 (m, 1H), 7.21 (dt, *J* = 8.1, 2.0 Hz, 1H), 6.35 (d, *J* = 7.7 Hz, 1H), 4.25–3.95 (m, 3H), 2.88 (t, *J* = 11.9 Hz, 2H), 2.00 (dd, *J* = 12.5, 2.4 Hz, 2H), 1.55–1.29 (m, 11H). ^13^C NMR (50 MHz, CDCl_3_) δ 165.03–160.03 (d, *J* = 250.0 Hz), 164.33 (d, *J* = 2.6 Hz), 154.70, 137.74 (d, *J* = 7.5 Hz), 135.49 (d, *J* = 10.1 Hz), 123.10 (d, *J* = 3.3 Hz), 119.06 (d, *J* = 24.8 Hz), 112.96 (d, *J* = 22.9 Hz), 79.84, 47.63, 42.72 (2C), 31.95 (2C), 28.40 (3C).

*tert-Butyl-4-(3-fluoro-4-methoxybenzamido)piperidine-1-carboxylate **5h**.* White solid, m.p. 132–134 °C, yield 44%. ^1^H NMR (200 MHz, CDCl_3_) δ 7.46 (d, *J* = 9.9 Hz, 2H), 6.88 (t, *J* = 8.2 Hz, 1H), 6.13 (d, *J* = 7.7 Hz, 2H), 4.12–3.95 (m, 3H), 3.85 (s, 3H), 2.93–2.71 (m, 2H), 1.92 (dd, *J* = 12.6, 2.6 Hz, 2H), 1.47–1.19 (m, 11H). ^13^C NMR (50 MHz, CDCl_3_) δ 165.31 (d, *J* = 2.0 Hz), 154.70–149.39 (d, *J* = 265.5 Hz), 154.31, 150.38 (d, *J* = 10.7 Hz), 127.26 (d, *J* = 5.5 Hz), 123.46 (d, *J* = 3.5 Hz), 115.05 (d, *J* = 19.7 Hz), 112.62 (d, *J* = 2.0 Hz), 79.70, 56.25, 47.32, 42.77 (2C), 32.09 (2C), 28.40 (3C).

*tert-Butyl-4-(2-nitro-4-(trifluoromethyl)benzamido)piperidine-1-carboxylate **5i**.* Pale yellow solid, m.p. 137–139 °C, yield 56%. ^1^H NMR (400 MHz, CDCl_3_) δ 8.32 (d, *J* = 6.2 Hz, 1H), 7.95–7.87 (m, 1H), 7.65 (dd, *J* = 7.5, 4.6 Hz, 1H), 6.13 (d, *J* = 68.3 Hz, 1H), 4.05 (d, *J* = 13.3 Hz, 3H), 2.90 (t, *J* = 11.5 Hz, 2H), 2.05 (d, *J* = 3.8 Hz, 2H), 1.50–1.27 (m, 11H). ^13^C NMR (101 MHz, CDCl_3_) δ 164.66, 154.67, 146.37, 136.00, 130.51–130.38 (m), 129.86 (d, *J* = 1.9 Hz), 123.73–121.02 (d, *J* = 273.7 Hz), 121.97–121.84 (m), 79.85, 47.73 (d, *J* = 5.1 Hz), 42.56 (2C), 31.52 (2C), 28.36 (3C). ^19^F NMR (376 MHz, CDCl_3_) δ -63.10.

*tert-Butyl-4-(1-naphthamido)piperidine-1-carboxylate **5j**.* White solid, m.p. 165–166 °C, yield 65%. ^1^H NMR (200 MHz, CDCl_3_) δ 8.29–8.18 (m, 1H), 7.95–7.80 (m, 2H), 7.62–7.34 (m, 4H), 6.03 (d, *J* = 7.8 Hz, 1H), 4.26–4.03 (m, 3H), 3.06–2.82 (m, 2H), 2.07 (dd, *J* = 12.7, 2.7 Hz, 2H), 1.57–1.29 (m, 11H). ^13^C NMR (50 MHz, CDCl_3_) δ 168.96, 154.70, 134.43, 133.65, 130.56, 130.03, 128.33, 127.13, 126.44, 125.19, 124.82, 124.69, 79.71, 47.27, 42.69 (2C), 32.05 (2C), 28.42 (3C).

#### 2.3.3. General Procedure for the Synthesis for Compounds **6a**–**j**

Derivatives **5a**–**j** (1.0 mmol) dissolved in methylene chloride were added to a reaction flask. Then, trifluoroacetic acid (3.0 mmol) was added and the reaction mixture was allowed to stir for two hours at room temperature. Complete conversion to the respective free amine was confirmed by TLC. Subsequently, the solvent was removed under reduced pressure and the crude obtained without purification was used for the subsequent step.

#### 2.3.4. General Procedure for the Synthesis for Compounds **7a**–**j**

2,3-Dichloro-1,4-naphthoquinone (1.0 mmol) and DIEA (3.0 mmol) dissolved in acetonitrile were added to the crude obtained in the previous synthetic step (**6a**–**j**). The mixture was stirred for 4 h at room temperature. Once the reaction time was complete, the solvent was evaporated, and the crude obtained was purified by a silica gel column using a 6:1 chloroform/acetone mixture as a mobile phase.

*2-Bromo-N-(1-(3-chloro-1,4-dioxo-1,4-dihydronaphthalen-2-yl)piperidin-4-yl)benzamide **7a**.* Purple solid, m.p. 197–198 °C, yield 37%. ^1^H NMR (400 MHz, CDCl_3_) δ 8.08 (dd, *J* = 7.4, 1.4 Hz, 1H, H7″), 7.98 (dd, *J* = 7.4, 1.5 Hz, 1H, H6″), 7.66 (pd, *J* = 7.4, 1.5 Hz, 2H, H5″ and H8″), 7.55 (dd, *J* = 8.0, 0.7 Hz, 1H, H6), 7.52 (dd, *J* = 7.6, 1.6 Hz, 1H, H3), 7.33 (td, *J* = 7.5, 0.9 Hz, 1H, H4), 7.25 (dd, *J* = 7.8, 1.6 Hz, 1H, H5), 5.98 (d, *J* = 7.9 Hz, 1H, NH), 4.36–4.21 (m, 1H, H1′), 3.81 (d, *J* = 13.7 Hz, 2H, H3′ and H5′), 3.51–3.37 (m, 2H, H3′ and H5′), 2.20 (dd, *J* = 12.6, 3.0 Hz, 2H, H2′ and H6′), 1.78 (ddd, *J* = 23.7, 11.6, 3.8 Hz, 2H, H2′ and H6′). ^13^C NMR (101 MHz, CDCl_3_) δ 181.96, 178.09, 166.94, 150.44, 137.66, 134.12, 133.38, 133.19, 131.61, 131.47, 131.36, 129.64, 127.66, 126.91, 126.64, 123.92, 119.20, 50.51, 46.97 (2C), 32.87 (2C). HRMS calcd. for (C_22_H_18_BrClN_2_O_3_ [M + H]^+^): 475.0242. Found: 475.0247.

*N-(1-(3-chloro-1,4-dioxo-1,4-dihydronaphthalen-2-yl)piperidin-4-yl)-4-nitrobenzamide **7b**.* Red solid, m.p. 190–192 °C, yield 48%. ^1^H NMR (400 MHz, CDCl_3_) δ 8.24 (d, *J* = 8.6 Hz, 2H), 8.07–8.03 (m, 1H), 7.99–7.91 (m, 3H), 7.70–7.60 (m, 2H), 6.38 (d, *J* = 7.7 Hz, 1H), 4.34–4.19 (m, 1H), 3.82 (d, *J* = 13.6 Hz, 2H), 3.41 (t, *J* = 11.8 Hz, 2H), 2.17 (d, *J* = 10.0 Hz, 2H), 1.79 (ddd, *J* = 23.8, 11.9, 3.8 Hz, 2H). ^13^C NMR (101 MHz, CDCl_3_) δ 181.88, 178.12, 164.90, 150.45, 149.63, 140.08, 134.19, 133.28, 131.55, 131.42, 128.21 (2C), 126.95, 126.62, 123.92, 123.83 (2C), 50.57(2C), 47.29, 32.99 (2C). HRMS calcd. for (C_22_H_18_ClN_3_O_5_ [M + H]^+^): 440.1008. Found: 440.1002.

*3-Chloro-N-(1-(3-chloro-1,4-dioxo-1,4-dihydronaphthalen-2-yl)piperidin-4-yl)benzamide **7c.*** Red solid, m.p. 129–131 °C, yield 61%. ^1^H NMR (400 MHz, CDCl_3_) δ 8.11–8.07 (m, 1H), 7.99 (dd, *J* = 7.3, 1.4 Hz, 1H), 7.76 (s, 1H), 7.72–7.65 (m, 3H), 7.48–7.42 (m, 1H), 7.36 (t, *J* = 7.8 Hz, 1H), 6.27 (d, *J* = 7.7 Hz, 1H), 4.37–4.17 (m, 1H), 3.84 (d, *J* = 13.6 Hz, 2H), 3.43 (t, *J* = 11.7 Hz, 2H), 2.17 (d, *J* = 10.3 Hz, 2H), 1.79 (qd, *J* = 11.9, 3.9 Hz, 2H). ^13^C NMR (101 MHz, CDCl_3_) δ 181.90, 178.09, 165.59, 150.44, 136.32, 134.77, 134.12, 133.19, 131.57, 131.45, 129.93, 127.31, 126.92, 126.61, 125.07, 123.82, 50.62 (2C), 46.98, 33.07 (2C). HRMS calcd. for (C_22_H_18_Cl_2_N_2_O_3_ [M + H]^+^): 429.0767. Found: 429.0762.

*N-(1-(3-chloro-1,4-dioxo-1,4-dihydronaphthalen-2-yl)piperidin-4-yl)-4-(chloromethyl)benzamide **7d.*** Purple solid, m.p. 208–210 °C, yield 55%. ^1^H NMR (400 MHz, CDCl_3_) δ 8.10 (dd, *J* = 7.4, 1.2 Hz, 1H), 8.03–7.98 (m, 1H), 7.77 (d, *J* = 8.2 Hz, 2H), 7.68 (pd, *J* = 7.3, 1.3 Hz, 2H), 7.45 (d, *J* = 8.1 Hz, 2H), 6.21 (d, *J* = 7.7 Hz, 1H), 4.60 (s, 2H), 4.36–4.19 (m, 1H), 3.84 (d, *J* = 13.5 Hz, 2H), 3.44 (t, *J* = 11.7 Hz, 2H), 2.18 (d, *J* = 12.4 Hz, 2H), 1.78 (qd, *J* = 12.0, 3.8 Hz, 2H). ^13^C NMR (101 MHz, CDCl_3_) δ 181.94, 178.08, 166.28, 150.45, 140.94, 134.48, 134.12, 133.19, 131.61, 131.47, 128.75, 127.39, 126.91, 126.63, 123.83, 50.64, 46.84, 45.35, 33.14, 30.92. HRMS calcd. for (C_23_H_20_Cl_2_N_2_O_3_ [M + H]^+^): 443.0924. Found: 443.0920.

*N-(1-(3-chloro-1,4-dioxo-1,4-dihydronaphthalen-2-yl)piperidin-4-yl)-3,5-dimethylbenzamide **7e.*** Red solid, m.p. 206–208 °C, yield 57%. ^1^H NMR (400 MHz, CDCl_3_) δ 8.11 (dd, *J* = 7.5, 1.3 Hz, 1H), 8.01 (dd, *J* = 7.4, 1.4 Hz, 1H), 7.68 (pd, *J* = 7.4, 1.5 Hz, 2H), 7.37 (s, 2H), 7.12 (s, 1H), 6.10 (d, *J* = 7.8 Hz, 1H), 4.36–4.18 (m, 1H), 3.84 (d, *J* = 13.6 Hz, 2H), 3.50–3.37 (m, 2H), 2.35 (s, 6H), 2.18 (d, *J* = 11.8 Hz, 2H), 1.78 (ddd, *J* = 23.9, 11.9, 3.9 Hz, 2H). ^13^C NMR (101 MHz, CDCl_3_) δ 181.96, 178.07, 167.25, 150.47, 138.33, 134.53, 134.10, 133.15, 131.63, 131.48, 126.90, 126.62, 124.65, 123.74, 50.67, 46.61, 33.20, 21.23. HRMS calcd. for (C_24_H_23_ClN_2_O_3_ [M + H]^+^): 423.1470. Found: 423.1470.

*N-(1-(3-chloro-1,4-dioxo-1,4-dihydronaphthalen-2-yl)piperidin-4-yl)-3,5-dinitrobenzamide **7f.*** Red solid, m.p. 223–225 °C, yield 29%. ^1^H NMR (400 MHz, CDCl_3_) δ 9.12–9.01 (m, 2H), 8.86 (dd, *J* = 6.3, 5.4 Hz, 1H), 8.71 (d, *J* = 7.8 Hz, 1H), 7.85 (t, *J* = 8.4 Hz, 1H), 7.78 (dd, *J* = 12.1, 5.7 Hz, 1H), 7.55–7.42 (m, 2H), 4.07 (s, 1H), 3.65 (d, *J* = 11.4 Hz, 2H), 3.20 (t, *J* = 12.1 Hz, 2H), 1.86 (d, *J* = 11.2 Hz, 2H), 1.78–1.61 (m, 2H). ^13^C NMR (101 MHz, CDCl_3_) δ 181.58, 177.69, 161.60, 150.39, 148.20, 137.98, 133.96, 133.05, 131.34, 131.29, 128.03, 126.71, 126.21, 122.95, 120.40, 50.63, 47.14, 32.39, 30.73. HRMS calcd. for (C_22_H_17_ClN_4_O_7_ [M + H]^+^): 485.0859. Found: 485.0859.

*3-Chloro-N-(1-(3-chloro-1,4-dioxo-1,4-dihydronaphthalen-2-yl)piperidin-4-yl)-5-fluorobenzamide **7g.*** Red solid, m.p. 200–202 °C, yield 42%. ^1^H NMR (400 MHz, CDCl_3_) δ 8.10–8.03 (m, 1H, H7″), 7.97 (dd, *J* = 7.3, 1.2 Hz, 1H, H6″), 7.65 (qd, *J* = 7.4, 6.1 Hz, 2H, H5″ and H8″), 7.51 (s, 1H, H2), 7.38 (d, *J* = 8.4 Hz, 1H, H6), 7.18 (d, *J* = 8.0 Hz, 1H, H4), 6.23 (d, *J* = 7.4 Hz, 1H, NH), 4.29–4.18 (m, 1H, H1′), 3.81 (d, *J* = 13.5 Hz, 2H, H3′ and H5′), 3.40 (t, *J* = 12.0 Hz, 2H, H3′ and H5′), 2.14 (d, *J* = 9.4 Hz, 2H, H2′ and H6′), 1.77 (dd, *J* = 20.3, 11.3 Hz, 2H, H2′ and H6′). ^13^C NMR (101 MHz, CDCl_3_) δ 181.89, 178.11, 164.37, 163.84, 161.34, 150.43, 137.80, 137.73, 135.65, 135.55, 134.15, 133.23, 131.58, 131.44, 126.93, 126.63, 123.95, 123.10, 123.07, 119.30, 119.05, 113.09, 112.87, 50.57, 47.18 (2C), 33.00 (2C). ^19^F NMR (376 MHz, CDCl_3_) δ -109.23 (1F). HRMS calcd. for (C_22_H_17_Cl_2_FN_2_O_3_ [M + H]^+^): 447.0673. Found: 447.0672.

*N-(1-(3-chloro-1,4-dioxo-1,4-dihydronaphthalen-2-yl)piperidin-4-yl)-3-fluoro-4-methoxybenzamide **7h.*** Red solid, m.p. 213–214 °C, yield 48%. ^1^H NMR (400 MHz, CDCl_3_) δ 8.08 (dd, *J* = 7.4, 1.4 Hz, 1H), 7.98 (dd, *J* = 7.4, 1.5 Hz, 1H), 7.65 (pd, *J* = 7.4, 1.5 Hz, 2H), 7.55–7.50 (m, 2H), 6.94 (t, *J* = 8.5 Hz, 1H), 6.03 (d, *J* = 7.8 Hz, 1H), 4.31–4.14 (m, 1H), 3.90 (s, 3H), 3.81 (d, *J* = 13.6 Hz, 2H), 3.46–3.34 (m, 2H), 2.20–2.07 (m, 2H), 1.74 (qd, *J* = 11.9, 3.9 Hz, 2H). ^13^C NMR (101 MHz, CDCl_3_) δ 181.95, 178.09, 165.32 (d, *J* = 1.8 Hz), 153.19–150.73 (d, *J* = 247.7 Hz), 150.56, 150.46, 134.12, 133.18, 131.54 (d, *J* = 14.2 Hz), 127.25 (d, *J* = 5.6 Hz), 126.77 (d, *J* = 28.7 Hz), 123.84, 123.38, 123.34, 115.10 (d, *J* = 19.6 Hz) 112.69, 56.30, 50.65, 46.85, 33.18. ^19^F NMR (376 MHz, CDCl_3_) δ -133.94. HRMS calcd. for (C_23_H_20_ClFN_2_O_4_ [M + H]^+^): 443.1168. Found: 443.1168.

*N-(1-(3-chloro-1,4-dioxo-1,4-dihydronaphthalen-2-yl)piperidin-4-yl)-2-nitro-4-(trifluoromethyl)benzamide **7i.*** Red solid, m.p. 169–171 °C, yield 13%. ^1^H NMR (400 MHz, CDCl_3_) δ 8.30 (s, 1H), 8.06–7.98 (m, 1H), 7.98–7.93 (m, 1H), 7.90 (d, *J* = 7.7 Hz, 1H), 7.72–7.59 (m, 3H), 6.12 (d, *J* = 7.8 Hz, 1H), 4.32–4.27 (m, 1H), 3.80 (d, *J* = 13.6 Hz, 2H), 3.42 (t, *J* = 11.7 Hz, 2H), 2.21 (d, *J* = 10.1 Hz, 2H), 1.76 (qd, *J* = 12.3, 3.5 Hz, 2H). ^13^C NMR (101 MHz, CDCl_3_) δ 181.85, 178.09, 164.71, 150.44, 146.37, 136.08–135.93 (m), 134.15, 133.55, 133.26, 133.20, 132.86, 132.52, 131.52, 131.41, 130.51 (dd, *J* = 6.6, 3.1 Hz), 129.87, 126.94, 126.59, 123.97, 121.97 (dd, *J* = 7.5, 3.5 Hz), 50.43, 47.29, 32.56. HRMS calcd. for (C_23_H_17_ClF_3_N_3_O_5_ [M + H]^+^): 508.0882. Found: 508.0878.

*N-(1-(3-chloro-1,4-dioxo-1,4-dihydronaphthalen-2-yl)piperidin-4-yl)-1-naphthamide **7j.*** Red solid, m.p. 212–214 °C, yield 34%. ^1^H NMR (400 MHz, CDCl_3_) δ 8.28 (d, *J* = 8.1 Hz, 1H), 8.10–8.07 (m, 1H), 8.01–7.98 (m, 1H), 7.89 (d, *J* = 8.2 Hz, 1H), 7.87–7.82 (m, 1H), 7.71–7.62 (m, 2H), 7.60–7.50 (m, 3H), 7.43 (dd, *J* = 8.1, 7.2 Hz, 1H), 6.12 (d, *J* = 7.9 Hz, 1H), 4.46–4.29 (m, 1H), 3.84 (d, *J* = 13.6 Hz, 2H), 3.51–3.38 (m, 2H), 2.30–2.19 (m, 2H), 1.80 (qd, *J* = 11.9, 3.9 Hz, 2H). ^13^C NMR (101 MHz, CDCl_3_) δ 181.93, 178.07, 169.01, 150.44, 134.42, 134.11, 133.68, 133.17, 131.59, 131.46, 130.64, 130.06, 128.37, 127.18, 126.90, 126.61, 126.47, 125.22, 124.90, 124.72, 123.82, 50.62, 46.82, 33.12. HRMS calcd. for (C_26_H_21_ClN_2_O_3_ [M + H]^+^): 445.1313. Found: 445.1310.

### 2.4. Trypanocidal Activity

#### 2.4.1. Trypanocidal Effect

Epimastigotes from *T. cruzi* strains NINOA and INC-5 were cultured in BHI (brain heart infusion broth, 89%) axenic medium, as reported in [26], at an initial concentration of 1 × 10^6^ parasites/mL, at 28 °C, supplemented with 10% foetal bovine serum and 1% sodium penicillin-streptomycin. Epimastigote growth was supervised by measuring the absorbance at 630 nm of the culture in an ELISA Epoch reader (Epoch 2 Microplate Spectrophotometer (BioTek Instruments, Inc., Winooski, VT, USA) daily. The assayed drugs were initially dissolved in DMSO at a concentration of 25 mM. This stock solution was then diluted with culture medium, ensuring that the final DMSO concentration did not exceed 0.4%. The control contained only 0.4% DMSO in the culture medium. Bzn was used as the reference compound, and epimastigote growth as the control. The concentrations of Bzn and each series of corresponding drug were monitored daily for five days. On the last day, the absorbance of the cultures was measured and related to the control. All measurements were carried out in triplicate. No toxic effect was attributable to DMSO. Since parasite growth is exponential, the data were analysed according to a first order equation, and the respective growth constant (k), corresponding to the slope, was calculated for controls and parasites treated with different drug concentrations. From these measurements, percentage growth inhibition (%GI) and half-maximal inhibitory concentration (IC_50_) values were calculated.

#### 2.4.2. Ex Vivo Evaluation of INC-5 and NINOA Strain Trypomastigotes

Those compounds with the lowest IC_50_ values in the epimastigote studies of each strain were evaluated in trypomastigotes. For this purpose, bloodstream trypomastigotes of INC-5 and NINOA strains were used for peritoneal injection into 6−8-week-old CD1 mice. The peak of parasitemia was achieved after 4–6 weeks, at which time parasitised blood samples were obtained by cardiac puncture using sodium heparin as an anticoagulant and diluted to a concentration of 1 × 10^6^ trypomastigotes/mL. 10 µL of each of the dilutions of the study compounds and Bzn, with these being placed in a 96-well plate, and 90 µL of infected blood was added, providing a final volume of 100 µL per well. The microplates were incubated at 4 °C for 24 h, and concentrations of 20 µM, 10 µM, and 5 µM of each compound were considered in these experiments. Each evaluation was performed in triplicate. Wells with untreated blood trypomastigotes were used as a negative lysis control and Bzn as a positive control. Then, the degree of lysis of trypomastigotes was determined by their reduction in number using an optical microscope according to the Brener–Pizzi method. For this purpose, 5 μL of blood was placed between a slide and a 13 × 13 coverslip. Mobile trypomastigotes were counted in 15 microscope fields at 40× magnification. Quantification of the degree of lysis for each case was expressed as a percentage considering the number of viable trypomastigotes with respect to the negative control [27]. The half-maximal lytic concentration (LC_50_) was determined by linear regression. The experiments were carried out in accordance with the recommendations and approval of the local Ethics and Research Committee (Approval number: ENCB/CEI/078/2020).

### 2.5. Cytotoxicity Assays

Cytotoxicity assays for the compounds selected for their trypanocidal activity were performed on J774 macrophage cells using the MTT method [28]. For this purpose, 50,000 cells/well resuspended in RPMI medium and 2% foetal bovine serum were seeded in a 96-well plate and incubated for 24 h at 37 °C. From the stock solutions of each of the compounds and Bzn, dilutions were made sequentially with PBS so that the final concentration in the well of each of these did not exceed 1% DMSO. After 24 h of incubation, the existing culture medium was replaced in the plate with fresh culture medium along with 5 mL each of the compound dilutions (100 to 1.25 μM) and Bzn to a final volume of 100 µL per well. This plate was then incubated in 5% CO_2_ for 24 h at 37 °C. The controls used were cells with medium as a positive control and cells without culture medium as a negative control. After the 24 h incubation, the morphology of the macrophages was observed under a microscope and then the culture medium was removed again and the MTT solution (5 mg/mL) was added. After 1 h incubation at 37 °C, the absorbance at 570 nm was recorded on an Elisa Epoch reader. The following formula was used to calculate the % cytotoxicity: %cytotoxicity = (100 − (mean number of cells with treatment/cells without treatment) × 100).

### 2.6. Docking

#### 2.6.1. Ligand Preparation

Synthesised compounds **2a**–**i** and **7a**–**j** were initially drawn in the open access software Marvin Sketch 18.10 and were then saved in 2D*.mol2* files. These 2D files were further-on energy minimised and converted to 3D.*mol2* format using the open access software Open Babel 2.4.1. Next, the .*mol2* file for each compound was processed using the software ACPYPE/AmberTools 17 to generate .*pdb* files and to prepare other files required to run molecular dynamics simulations in a later stage of the in silico analysis. Lastly, *pdb* files were converted to.pdbqt format utilizing MGLTools 1.5.6 executable *prepare_ligand4.py.* Along with these compounds, mepacrine, a known TR inhibitor, and three lead structures from Vera et al. [21], a previous study with naphthoquinones, were prepared for molecular docking.

#### 2.6.2. Predicted Bindings Site

The Site Finder tool of the MOE software was used to predict the binding sites for the tested compounds. Residues present at the site were considered to locate the docking Grid Box. PyMol software was used to locate the Grid Box centre using the *centerofmass* function for residues present at the predicted site.

#### 2.6.3. Protein Preparation

Protein crystal was obtained from the Protein Data Bank (PDB) in *.pdb* format with access code 1BZL. The protein was prepared for molecular docking using open-source software. Initially, the proteins were cleaned using the USCFChimera 1.12 software, and any atom that was co-crystalised in structure, bound ligand, and other ions were removed from structure. The Dock Prep tool was then used for each of the cleaned proteins to add hydrogens and any charges to amino acids to prepare for molecular docking. The prepared crystal was exported in .*pdb* format for further use.

#### 2.6.4. Molecular Docking

Using the open source software Auto Dock Tools, the receptor structure was converted to *.pdbqt* format and the ligands were set up to be docked at the Z-site with the centre at *X*, *Y*, and *Z*-coordinates 41.825, 4.351, and −28.343, respectively, with 24 × 24 × 24 Å dimensions in each axis. The AutoDock Vina docking protocol was used with a spacing of 1 Å, generating nine models for each docked compound. All of the compounds were analysed to determine the interactions present in the receptor–ligand complex obtained by molecular docking analysis. The interactions were determined using an online server, the Protein–Ligand Interaction Profiler (PLIP). The interaction profiles for the tested compounds were compared to the interactions observed for the naphthoquinones previously reported by our group: **NQ-d**, **NQ-g**, and **NQ-h**.

### 2.7. Trypanothione Reductase Inhibition Assay

The inhibition of *Tc*TR activity by **7j** was evaluated by monitoring the trypanothione disulphide (TS_2_)-dependent NADPH oxidation by using the assay described before [29]. The reaction mixture contained assay buffer (40 mM HEPES pH 7.4, 1 mM EDTA), recombinant *Tc*TR (88-168 ng/mL), NADPH (the concentration depended on experiment), and different **7j** concentrations when required, in a volume of 500 μL. The mixture was incubated at 37 °C for 3 min, and TS_2_ was added to start the reaction. The absorbance at 340 nm was recorded in real time for 10 min, and the activity was calculated from the slope of the curves as μmol of NADPH oxidised per min (e^340^ = 6.22 mM^−1^ cm^−1^).

## 3. Results and Discussion

### 3.1. Chemistry

**Scheme 1:**
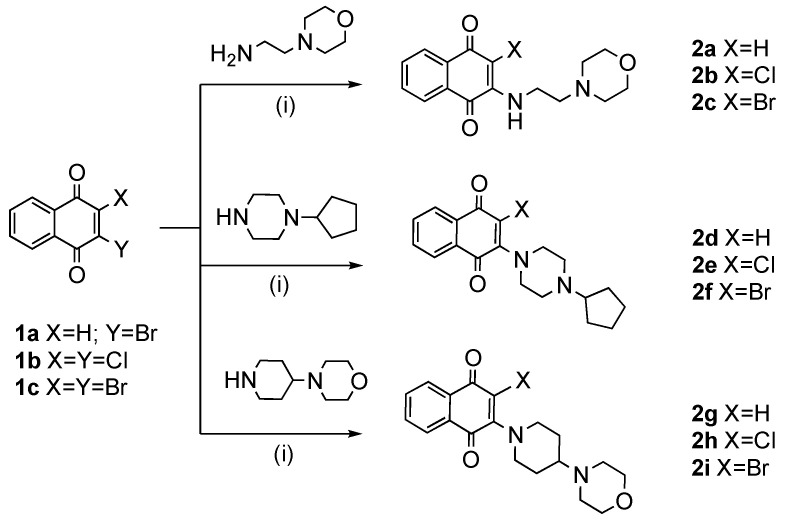
Synthesis of quinone derivatives belong to **Series I**. *Reagents and conditions:* (i) DIEA, CH_3_CN, r.t. 4 h, 18–86%.

The synthesis of derivatives from **Series I** (**2a**–**i**) was conducted according to a previously reported procedure [12,27,30], and as shown in Scheme 1, using mono- or dihalo-naphthoquinones **1a**–**c** as starting material. The nucleophilic substitution of **1a**–**c** with 2-morpholinoethanamine, 1-cyclopentylpiperazine, or 4-(piperidin-4-yl)morpholine in basic medium, with acetonitrile as a solvent at room temperature, yielded the quinone derivatives **2a**–**i** (18–86%).

**Scheme 2:**
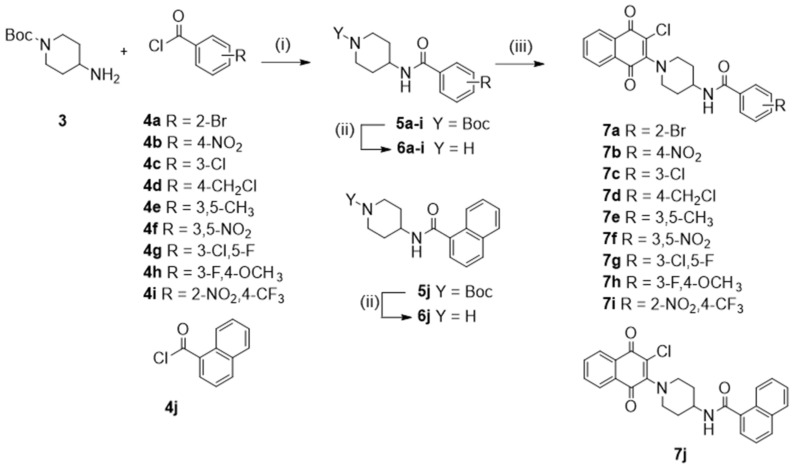
Synthesis of quinone derivatives belong to **Series II**. *Reagents and conditions:* (i) Et_3_N, THF, 3 h, r.t., 42–72% (ii) TFA, CH_2_Cl_2_, 2 h, r.t. (iii) **1b**, DIEA, CH_3_CN, r.t. 4 h, 13–61%.

The derivatives from **Series II** (**7a**–**j**) were obtained through the synthetic pathway shown in Scheme 2. First, *N*-(piperidin-4-yl)benzamides **6a**–**j** were obtained by a nucleophilic substitution reaction between *tert*-butyl-4-aminopiperidin-1-carboxilate **3** and the respective acyl chlorides **4a**–**j** in triethylamine (Et_3_N) to yield the piperidine amide derivatives **5a**–**j** [25]. Then, **5a**–**j** were treated with trifluoracetic acid (TFA) and dichloromethane as a solvent at room temperature to yield the amides **6a**–**j**, which, without further purification, reacted with **1b**, using acetonitrile as a solvent and *N,N*-diisopropylethylamine (DIEA) as a base, yielding the target compounds **7a**–**j**. All of the synthetised compounds were purified by column chromatography, and their structures were established based on their spectroscopic properties (IR, MS, ^1^H NMR, and ^13^C NMR, see the Experimental Section and Supplementary Information).

### 3.2. Trypanocidal Effects on Epimastigote and Trypomastigote Strains

In the trypanocidal evaluation for the synthesised amino naphthoquinone derivatives **2a**–**i** and **7a**–**j**, the growth inhibition (GI) of the epimastigote strains NINOA and INC-5 was evaluated (%GI). In these assays, Bzn was used as the reference drug because it was approved in 2017 by the United States Food and Drugs Administration (FDA) for the treatment of Chagas disease [21,22]. The results showed that all of the compounds had a %GI value greater than 70% in both strains using a concentration of the compounds at 10 μM (Table S1, Supporting Information). Therefore, based on the results, dose-dependent assays were carried out using several concentrations (0.625–5.0 μM) to determine the half-maximal inhibitory concentration (IC_50_) for these quinones in each strain and for the reference drug.

The IC_50_ values are shown in Table 1. The results showed that most compounds induced greater inhibitory activity than Bzn in both Mexican epimastigote strains. Compounds **2e** and **7j** exhibited the lowest IC_50_ values for the NINOA and INC-5 strains (0.43 µM for both strains for **2e** and 0.19 µM and 0.92 µM for **7j**). From a chemical viewpoint, a simple structure–activity relationship (SAR) for the compounds of **Series I**, showed that the most potent compounds in both strains had the cyclopentyl-piperazine fragment, which indicated that this nitrogen ring is an interesting scaffold to consider in new trypanocidal agents. Likewise, regarding the effect of the halogen atom at C-2 on the naphthoquinone core, a chlorine atom and bromine atom increases the parasiticidal activity on the NINOA strain and INC-5 strains, respectively. This evidence confirms our previous results regarding the importance of a halogen atom in this position [27]. Most of the **Series II** compounds were more potent than the **Series I** compounds. In the NINOA strain, compounds **7b**, **7c**, **7h,** and **7j** had IC_50_ values lower than 1.0 µM (0.77, 0.52, 0.84, and 0.43, respectively). However, for the INC-5 strain, the best results were obtained for compounds **7c**, **7h**, and **7j**, with the latter being the only one that had an IC_50_ value lower than 1.0 µM. SAR analysis for *N*-(piperidin-4-yl)benzamide quinones was not possible because the trypanocidal effect was heterogeneous. However, considering that the most active compound was **7j**, it appeared that a bulky group in the amide fragment (a naphthalene ring) was suitable to increase the activity in both strains.

Because these compounds had good activity on epimastigote strains, we decided to conduct our evaluation on the trypomastigote forms. Subsequently, for compounds with an IC_50_ < 3.0 µM in the epimastigote strains, their capacity for cell lysis of trypomastigote was evaluated (Table 1). In these assays, compounds **7b**, **7c**, **7f**, **7h,** and **7i** showed greater potency than Bzn on NINOA, whereas on INC-5, the compound with the highest activity was **7j**, with a LC_50_ = 59.73 µM. The significance of these results relates to the fact that trypomastigotes are the infective flagellated form of the parasite in the blood and are responsible for the acute and chronic stages of Chagas disease in mammalian hosts [31]. Epimastigotes are used for primary screening because their replication is extracellular and they are easy to handle and maintain in the laboratory [32].

### 3.3. Cytotoxicity in Murine Macrophages

The cytotoxic effect against J774 murine macrophages was evaluated for compounds with the highest activity on trypomastigote. Derivatives **7c**, **7f**, **7h,** and **7j** showed moderate cytotoxicity values (IC_50_ = ~25 µM; Table 2). With these data, it was possible to determine the selectivity index (SI) against the epimastigote and trypomastigote strains (Table 2). The SI values were higher for the epimastigote than trypomastigote form for these quinone derivatives. However, **7j** was more selective than Bzn on epimastigotes and was most selective on INC-5 trypomastigote compared with the other compounds in the study.

Once the in vitro characterization was conducted, an in silico study was developed on *Tc*TR to explore this putative target in relation to the trypanocidal effect of the compounds.

### 3.4. Molecular Docking Studies

**Figure 2:**
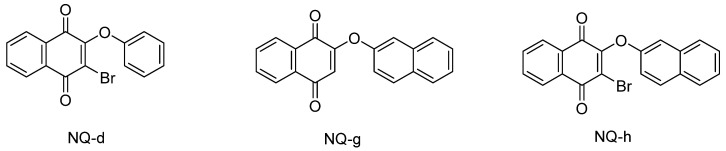
Chemical structures of *Tc*TR inhibitors used as controls.

TR is one of the most important enzymes for the survival of parasites because it participates in redox metabolism, catalysing the reduction of trypanothione disulphide (TS_2_) to trypanothione (TSH) [22,33,34]. Furthermore, it is considered an attractive target for the search of new anti-Chagasic agents since this enzyme is absent in mammals [33,34]. Additionally, previous studies have shown that naphthoquinones are potential TR inhibitors [21]. Thus, an in silico docking study was conducted to assess the possible inhibitory interactions of 1,4-naphthoquinone derivatives (**Series I** and **II**) on *Tc*TR. **NQ-d**, **NQ-g,** and **NQ-h,** shown in Figure 2, were considered control compounds for our molecular docking studies because these *Tc*TR inhibitors were previously reported by our research group [21].

**Figure 3:**
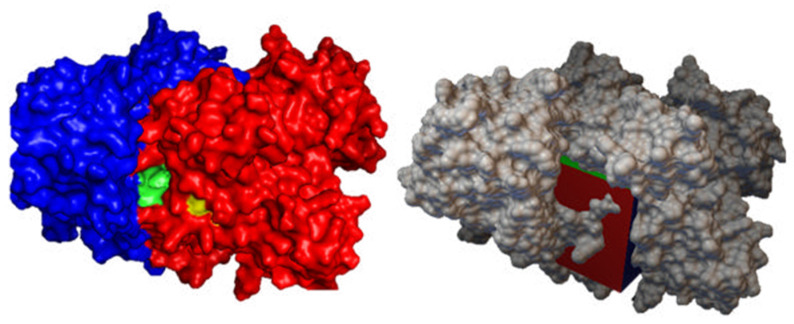
Visual representation of the “Z-site”: chain A (blue), chain B (red), grid box (red-green-blue cube).

The synthesised naphthoquinones were docked on a hydrophobic region of *Tc*TR named the “Z-site”, which was previously defined by el-Waer et al. [35]. In that work, the Z-site was suggested as a relevant binding pocket for TR inhibitors, and it is located at the innermost part of the dimer interphase and the nicotinamide adenine dinucleotide phosphate-binding site (Figure 3 and Table 3). The highest free energy of binding (FEB) values for the naphthoquinones are shown in Table 4; these scores were used to compare all of the compounds with the scores for **NQ-d**, **NQ-g**, and **NQ-h**. As shown in Table 4, nine quinone derivatives belonging to **Series II** (**7a**–**7j**, except **7c**) had a FEB value with a higher affinity for *Tc*TR than **NQ-g**. The remaining naphthoquinones from **Series I** (**2a**–**2i**) were predicted to have an FEB value with lower affinity than the control compounds. The difference in binding energies within the compounds from both **Series I** and **II** was minimal, which suggests that most of the predicted binding energies could be attributed to the naphthoquinone scaffold. The quinones from **Series I** with less affinity for *Tc*TR were **2a**–**c**, with a slightly flexible amino alkyl chain at C-3 of the naphthoquinone ring, suggesting that this flexibility was insufficient to attain a favourable position at the binding site. The other quinones of **Series I** (**2d**–**f** and **2g**–**i**) had similar FEB values, both with a bicycle substituent: a piperazinyl-cyclopentyl or piperidyl-morpholinyl ring, with the latter having a slightly better affinity. For the quinones from **Series II**, a slight tendency was observed when the phenyl ring was substituted at position 4; **7b**, **7d**, **7h**, and **7i** had the highest FEB values. A clear difference may be attributed in **7j,** as the substituent is a naphthyl group and not a phenyl group. This naphthyl fragment was present in two of the three lead quinones considered to be controls, **NQ-g** and **NQ-h**, which further supports the relevance of large aromatic groups favouring interactions with the binding site.

To include compounds that may have a higher probability of behaving as *Tc*TR inhibitors, all compounds with an equal or higher FEB score than the controls were considered for further analyses to determine their interaction profile.

**Figure 4:**
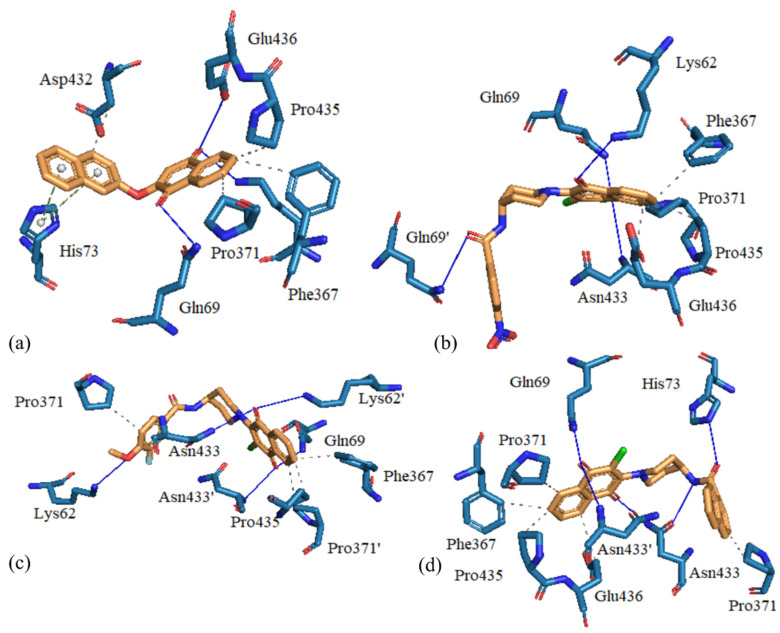
Binding modes on the Z-site of (**a**) **NQ-q**, (**b**) **7b**, (**c**) **7h,** and (**d**) **7j**.

Considering the highest values of FEB compared with **NQ-g**, an exhaustive study on the interaction profiles using nine models generated by VINA analysis for **Series II** quinones was conducted. Full details of the interactions observed between these quinones at the binding site are shown in Table 5. Hydrophobic interactions (**HI**), hydrogen bonds (**HB**), π-cation interactions (**π-c**), π-stacking (**π-s**), salt bridges (**SB**), and halogen interactions (**HalB**) were observed. Full details about the interaction profiles for each quinone are shown in Table S2. Interaction profiles observed for the predicted binding site for **NQ-g** (Figure 4) were used to compare with the tested compounds **2a-7j** to assess their potential as *Tc*TR inhibitors. As shown in Table S2, there were a few common residue interactions shared between **NQ-d**, **NQ-g**, and **NQ-h** and the tested compounds; of these, the most represented were Gln69′, Gln69, and Asn433.

The naphthoquinones that showed the most shared interactions with **NQ-g** on the predicted *Tc*TR-binding site were **7b**, **7d**, **7i**, **7h**, and **7j** (all had FEB values > 9.5 kcal/mol), which shared at least five out the eight amino acids involved in these interactions (Phe367, Pro371′, Pro435′, Gln69, and Glu436) (Figure 4).

### 3.5. Evaluation of ***7j*** on TcTR

**Figure 5:**
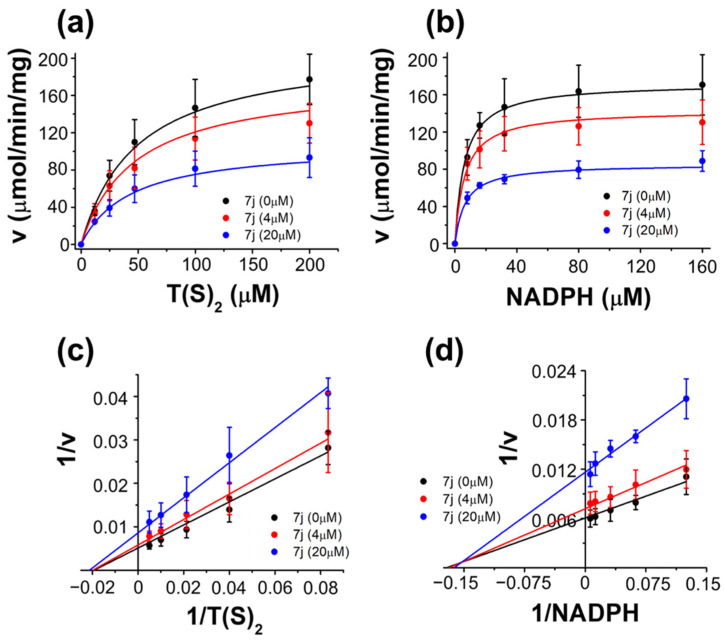
Kinetic behaviour of *Tc*TR in the presence of **7j**. Panel (**a**), the concentration of TS_2_ was varied at different concentrations of **7j** (0–20 µM) at NADPH fixed (160 µM). Panel (**b**), the concentration of NADPH was varied at different concentrations of **7j** (0–20 µM) at TS_2_ fixed (200 µM). Plots in panels (**a**,**b**) were obtained by a non-linear global fit to Equation (1) using Origin 8 software. Panels (**c**,**d**) show the Lineweaver−Burk plots of the kinetic data fitted to the linear form of Equation (1) using Origin 8 software. Data are the average of three independent enzyme preparations (±SD).

Considering the results obtained regarding the parasiticidal effect on epimastigotes and trypomastigotes of these naphthoquinone derivatives, in addition to their cytotoxicity on J774 and the docking studies on TR, we conducted kinetic assays on *Tc*TR for compound **7j**. To determine the type of enzyme inhibition, a study was carried out at different substrate concentrations, and the kinetic parameters of the enzyme were calculated using Lineweaver–Burk plots (Figure 5).

**7j** behaved as a simple mixed-type inhibitor with respect to both substrates, TS_2_ and NADPH, according to the patterns of intersecting lines in the double reciprocal plots (Figure 5c,d). Its most important effect was a decrease in Vmax (Figure 5a,b); the Km values remained highly similar, since the alpha value in the mixed-type inhibitor equation (Equation (1)) was close to 1 (Table 6). The kinetic data suggested that **7j** and substrates bind to different sites on the enzyme, which was consistent with the docking results for this naphthoquinone as well as with our previous results for **NQ-g** [21].

(1) $$v=\frac{Vm\left[S\right]}{Ks\left(1+\frac{\left[I\right]}{Ki}\right)+\left[S\right]\left(1+\frac{\left[I\right]}{\alpha Ki}\right)}$$

### 3.6. Calculated Physicochemical Properties

**Figure 6:**
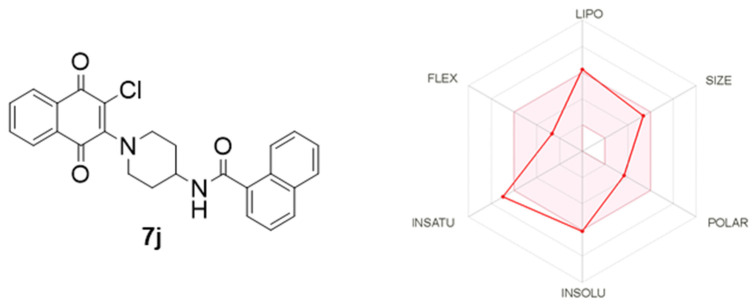
The bioavailability radar plot of **7j**. The pink zone represents the range of the values for optimal oral bioavailability property, and the predicted properties were displayed as red lines. The radar was generated by SwissADME (http://www.swissadme.ch/index.php, accessed on 27 January 2022).

Using the SwissADME web server (http://www.swissadme.ch/index.php, accessed on 27 January 2022), compound **7j** was analysed to predict its drug-likeness properties, such as its pharmacokinetic (absorption, distribution, metabolism, excretion, and ADME) and pharmacodynamic (e.g., toxicological) profiles. This is because these properties are used to guide rational drug design and to optimise a lead compound into a successful preclinical one [36]. ADME and toxicity properties are determined by some important chemical descriptors, such as the polar surface area (PSA) and molecular weight (MW) of the molecules, which are useful for predicting the oral absorption of drugs. Thus, to assess these properties and predict good oral bioavailability, there are two sets of rules, the Lipinski rule and the Veber rule, which are used for these purposes [37,38]. The Lipinski rule states that an orally bioavailable molecule must not fail to meet the following four criteria: ≤5 hydrogen bond donors (HBDs); ≤10 hydrogen bond acceptors (HBAs); MW < 500; and log *p*-value < 5. Additionally, Veber et al. established the role of PSA and the number of rotational bonds as criteria for estimating oral bioavailability. Veber’s rule states that for the compound to be orally bioavailable, it must have a PSA ≤ 140 Å, ≤10 rotational bonds (NRB) or ≤ 12 total HBDs, and HBAs and ≤ 10 rotational bonds. As shown in Table 7, compound **7j** met the criteria described by Lipinski and Veber; therefore, it is expected to have good oral bioavailability. This is also depicted in the bioavailability radar plot, which showed that **7j** falls within the desired range (pink region) of five parameters from the six parameters used for oral absorption prediction: flexibility, lipophilicity, solubility, size, and polarity, while it lies in the undesirable area of saturation (Figure 6), confirming its good oral bioavailability.

## 4. Conclusions

In this study, two series of amino naphthoquinones were synthesised and evaluated in vitro and ex vivo against the epimastigote and trypomastigote forms of two Mexican *T. cruzi* strains. The trypanocidal activity of these compounds against both forms was, in some cases, better than Bzn. The most promising naphthoquinone derivative is compound **7j** due to its higher trypanocidal activity on epimastigotes (INC-5 and NINOA strains) and trypomastigotes (NINOA strain) and its higher SI values against epimastigote forms compared to Bzn. Molecular docking studies and experimental kinetics assays showed that **7j** binds with and behaved as a mixed-type inhibitor of *Tc*TR, which was consistent with the interaction of **7j** in a site that was not a substrate-binding site. Thus, *Tc*TR can be a possible target of **7j** in the parasite and may contribute to the compound’s trypanocidal effect. On the other hand, predictions regarding ADME properties indicated that **7j** should have good oral bioavailability. Therefore, in this work, we have expanded the chemical library of trypanocidal compounds and validated the usefulness of naphthoquinone scaffolds as well as the incorporation of a nitrogenated fragment as a strategy for the development of anti-Chagasic agents.

## Figures and Tables

**Table 1:** Effect of culture growth of amino-naphthoquinone derivatives on *T. cruzi* epimastigote and trypomastigote forms.

Compound	Epi NINOA IC_50_ (µM) ^a,b^	Epi INC-5 IC_50_ (µM) ^a,b^	Trypo NINOA LC_50_ (µM) ^a,c^	Trypo INC-5 LC_50_ (µM) ^a,c^
**2a**	1.10 ± 0.12	3.06 ± 0.01	>100	>100
**2b**	1.10 ± 0.05	2.98 ± 0.2	>100	>100
**2c**	1.14 ± 0.06	2.22 ± 0.04	>100	>100
**2d**	1.12 ± 0.04	5.04 ± 0.26	>100	87.34 ± 5.32
**2e**	0.43 ± 0.15	0.19 ± 0.07	>100	>100
**2f**	1.15 ± 0.09	1.46 ± 0.03	97.86 ± 2.13	>100
**2g**	7.05 ± 0.95	8.45 ± 0.35	NT ^d^	NT ^d^
**2h**	6.40 ± 0.02	6.60 ± 0.08	NT ^d^	NT ^d^
**2i**	10.1 ± 0.05	1.74 ± 0.02	NT ^d^	NT ^d^
**7a**	6.41 ± 0.02	2.46 ± 0.08	NT ^d^	NT ^d^
**7b**	0.77 ± 0.21	2.23 ± 0.06	80.32 ± 4.03	>100
**7c**	0.52 ± 0.04	1.14 ± 0.07	68.35 ± 6.30	>100
**7d**	1.25 ± 0.06	2.30 ± 0.11	>100	>100
**7e**	4.45 ± 0.06	5.60 ± 0.03	NT ^d^	NT ^d^
**7f**	0.99 ± 0.01	1.84 ± 0.06	70.86 ± 7.31	98.56 ± 8.2
**7g**	3.55 ± 0.02	2.57 ± 0.04	>100	>100
**7h**	0.84 ± 0.11	1.35 ± 0.01	78.57 ± 3.91	94.65 ± 5.21
**7i**	1.09 ± 0.01	3.35 ± 0.05	90.45 ± 6.58	>100
**7j**	0.43 ± 0.03	0.92 ± 0.01	98.06 ± 8.9	59.73 ± 3.72
**Bzn**	8.21 ± 1.80	42.3 ± 5.80	>100	85.62 ± 4.23

^a^ The results are means of three independent experiments; ^b^ half-maximal inhibitory concentration on epimastigotes of *T. cruzi*: INC-5 and NINOA strains; ^c^ half-maximal lytic concentration of aryloxy naphthoquinones on trypomastigotes of *T. cruzi* strains: INC-5 and NINOA strains; ^d^ NT = not tested.

**Table 2:** IC_50_ values on J774 cells and SI values for selected compounds.

Compound	J774 IC_50_ (µM) ^a^	NINOA Epi SI ^b^	INC-5 Epi SI ^b^	NINOA Trypo SI ^b^	INC-5 Trypo SI ^b^
**7c**	25.0	48.0	21.9	0.37	ND ^c^
**7f**	25.0	25.2	13.6	0.32	0.25
**7h**	22.0	26.2	16.3	0.28	0.23
**7j**	33.0	76.7	35.9	0.34	0.55
**Bzn**	352	42.9	8.38	2.36	4.11

^a^ The results are means of three independent experiments; ^b^ SI value = IC_50_ values on J774 cells/IC_50_ or LC_50_ *T. cruzi*; ^c^ ND = not determined.

**Table 3:** Residues present at the “Z-site” *Tc*TR-binding site.

Putative Binding Z-Site	
**Chain A**	Lys62 Leu63 Val65 Thr66 Gln69 Tyr70 His73 Glu76 Gln242 Phe367 Ser368 Ile369 Pro370 Pro371 Pro398 Leu399 Met400 His401 Lys409 Thr410 Phe411 Leu430 Gly431 Asp432 Asn433 Pro435 Glu436 Pro462 Thr463 Ser464
**Chain B**	Lys62 Leu63 Val65 Thr66 Gln69 Tyr70 His73 Glu76 Phe367 Ser368 Ile369 Pro370 Pro371 Pro398 Leu399 Met400 His401 Phe411 Gly431 Asp432 Asn433 Pro435 Glu436 Pro462 Thr463 Ser464

**Table 4:** Free energy of binding of 1,4-naphthoquinone derivatives on the Z-site.

Compound	FEB TR (Kcal/Mol)	Compound	FEB TR (Kcal/Mol)
**2a**	−7.2	**7c**	−8.2
**2b**	−7.5	**7d**	−9.5
**2c**	−7.4	**7e**	−8.5
**2d**	−7.8	**7f**	−8.9
**2e**	−7.7	**7g**	−8.7
**2f**	−7.8	**7h**	−9.7
**2g**	−7.7	**7i**	−10.5
**2h**	−7.8	**7j**	−9.5
**2i**	−7.8	**NQ-d**	−7.8
**7a**	−8.8	**NQ-g**	−8.4
**7b**	−9.7	**NQ-h**	−8.0

**Table 5:** Interactions among **Series II** compounds at the *Tc*TR-binding site.

Compound	Interactions
**7a**	**HI**: Gln69, Glu72, Phe367, Pro435, Glu436. **HB**: Gln69, Asn433. **HalB**: Asn433.
**7b**	**HI**: Phe367, Pro371, Pro435, Glu436. **HB**: Lys62, Gln69, Asn433.
**7d**	**HI**: Phe367, Pro371, Pro435. **HB**: Lys62, Gln69, Asn433.
**7e**	**HI**: Gln69, Met400, Asp432. **HB**: Lys62, Asn433, Glu436
**7f**	**HI**: Phe367, Pro371, Asp432, Pro435, Glu436. **HB**: Gln69, Asn433.
**7g**	**HI**: Thr66, Gln69, Glu72, Pro371. **HB**: Lys62, Tyr70, His73, His401. **Π-c: His73**.
**7h**	**HI**: Phe367, Pro371, Pro435. **HB**: Lys62, Gln69, Asn433.
**7i**	**HI**: Phe367, Pro371, Asp432, Pro435, Glu436. **HB**: Lys62, Gln69, Asn433. **HalB**: Glu436.
**7j**	**HI**: Phe367, Pro371, Pro435, Glu436. **HB**: Gln69, His73, Asn433.
**NQ-d**	**HI**: Thr66, Gln69, Tyr70, Phe367, Pro371, Pro435, Glu436. **HB**. Gln69, Asn433. **Π-s**: His73.
**NQ-g**	**HI**: Phe367, Pro371, Asp432, Pro435. **HB**: Lys62, Gln69, Glu436. **Π-s**: His73.
**NQ-h**	**HI**: Phe367, Pro371, Asp432, Pro435. **HB**: Asp432.

**Table 6:** Kinetic parameters of the mixed-type inhibition of *Tc*TR by **7j**.

**Kinetic Parameter**	**Value**
*Vmax* (µmol/min/mg) ^a^	215 ± 38
*Km* TS_2_ (µM) ^a^	50 ± 4
*Km* NADPH (µM) ^a^	6.0 ± 0.2
*Ki***7j** vs. TS_2_ (µM) ^a^	28 ± 4.5
*Ki* **7j** vs. NADPH (µM) ^a^	18 ± 3.5
α TS_2_ ^b^	0.77
α NADPH ^b^	1.12

^a^ Values were estimated by a global nonlinear regression of data in Figure 5 using Equation (1); ^b^ Values were estimated by replots of slope and 1/v axis intercept vs. **7j**.

**Table 7:** Molecular properties of **7j**.

Compound	MW (Da)	HBA	HBD	cLogP	TPSA (A^2^)	NRB
**Desirable value**	≤500	≤10	≤5	≤5	≤140	≤10
**7j**	444.91	3	1	3.99	66.48	4

MW: molecular weight; HBA: number of hydrogen bond acceptors; HBD: number of hydrogen bond donors; cLogP: consensus Log P value; TPSA: topological polar surface; NRB: number of rotatable bonds.

## Data Availability

Not applicable.

## Supplementary Materials

The following supporting information can be downloaded at: https://www.mdpi.com/article/10.3390/pharmaceutics14061121/s1, ^1^H, ^13^C NMR, and HRMS of selected compounds; Table S1: Effect of amino naphthoquinone derivatives upon culture growth of *T. cruzi* epimastigote forms expressed as % growth inhibition at 10 μM; Table S2: Protein–ligand interaction profiles for naphthoquinones and controls.
