# Supplementary material for: New Amino Naphthoquinone Derivatives as Anti-Trypanosoma cruzi Agents Targeting Trypanothione Reductase
Source: Pharmaceutics. 2022 May 25;14(6):1121. doi: 10.3390/pharmaceutics14061121 (PMC9228152; doi:10.3390/pharmaceutics14061121)
Supplement: Supplementary file 1 [file pharmaceutics-14-01121-s001.zip › pharmaceutics-1720683-supplementary.pdf]

Supporting Information

New amino naphthoquinone derivatives as anti-*Trypanosoma cruzi* agents targeting trypanothione reductase

Christian Espinosa-Bustos[1], Mariana Ortiz Pérez[2], Alonzo Gonzalez-Gonzalez[3], Ana María Zarate[4], Gildardo Rivera[3], Javier A. Belmont-Díaz[5], Emma Saavedra[5], Mauricio A. Cuellar,[6] Karina Vázquez*[2] Cristian O. Salas*[4]

[1] Departamento de Farmacia, Facultad de Química y de Farmacia, Pontificia Universidad Católica de Chile, Vicuña Mackenna 4860, Santiago 702843, Chile.

[2] Departamento de Parasitología, Facultad de Medicina Veterinaria y Zootecnia, Universidad Autónoma de Nuevo León, Francisco Villa 20, Gral. Escobedo 66054, México.

[3] Laboratorio de Biotecnología Farmacéutica, Centro de Biotecnología Genómica, Instituto Politécnico Nacional, Boulevard del Maestro s/n, Reynosa 88710, México.

[4] Departamento de Química Orgánica, Facultad de Química y de Farmacia, Pontificia Universidad Católica de Chile, Vicuña Mackenna 4860, Macul, Santiago 702843, Chile.

[5] Departamento de Bioquímica, Instituto Nacional de Cardiología Ignacio Chávez, México City 14080, México.

[6] Centro de Investigación Farmacopea Chilena, Escuela de Química y Farmacia, Facultad de Farmacia, Universidad de Valparaíso, Av. Gran Bretaña 1093, Valparaíso 2340000, Chile.

Corresponding authors

Karina Vázquez and Cristian O. Salas

Email: karina.vazquezcn@uanl.edu.mx (K.V.); cosalas@uc.cl (C.O.S.)

Index

^{1}H and ^{13}C NMR of selected compounds ……………………………………………pag 02-17

HRMS of selected compounds…………………………………………….……….….. pag 18-26

Table S1. Effect of amino naphthoquinone derivatives upon culture growth of *T. cruzi* epimastigote forms expressed as % growth inhibition at 10 μM…………………. pag 27

Table S2. Protein-ligand interaction profiles for naphthoquinones and controls.………………………………………………………………………….pag 28-29

^{1}H NMR for 2c

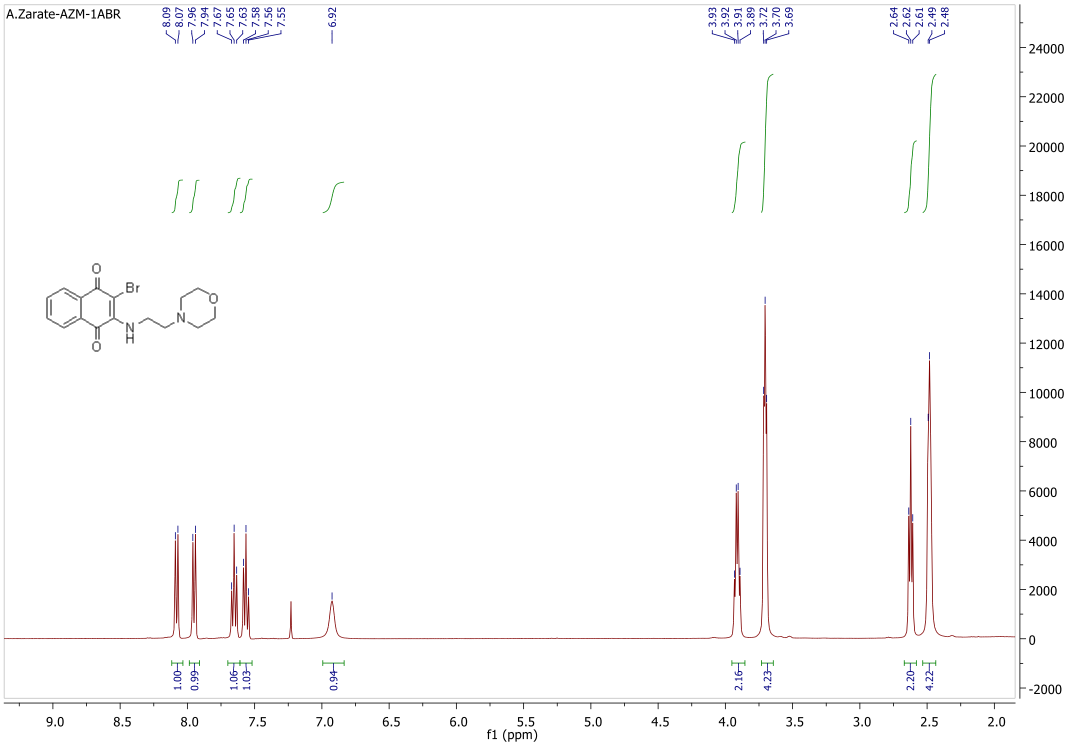

^{13}C NMR for 2c

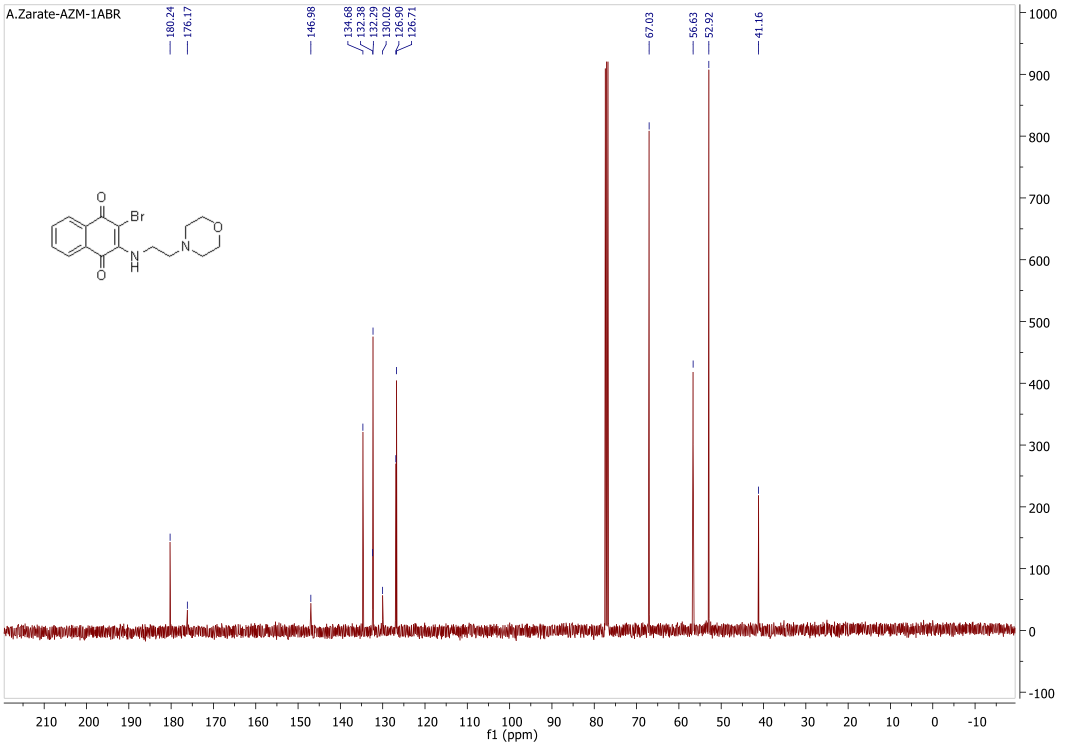

^{1}H NMR for 2d

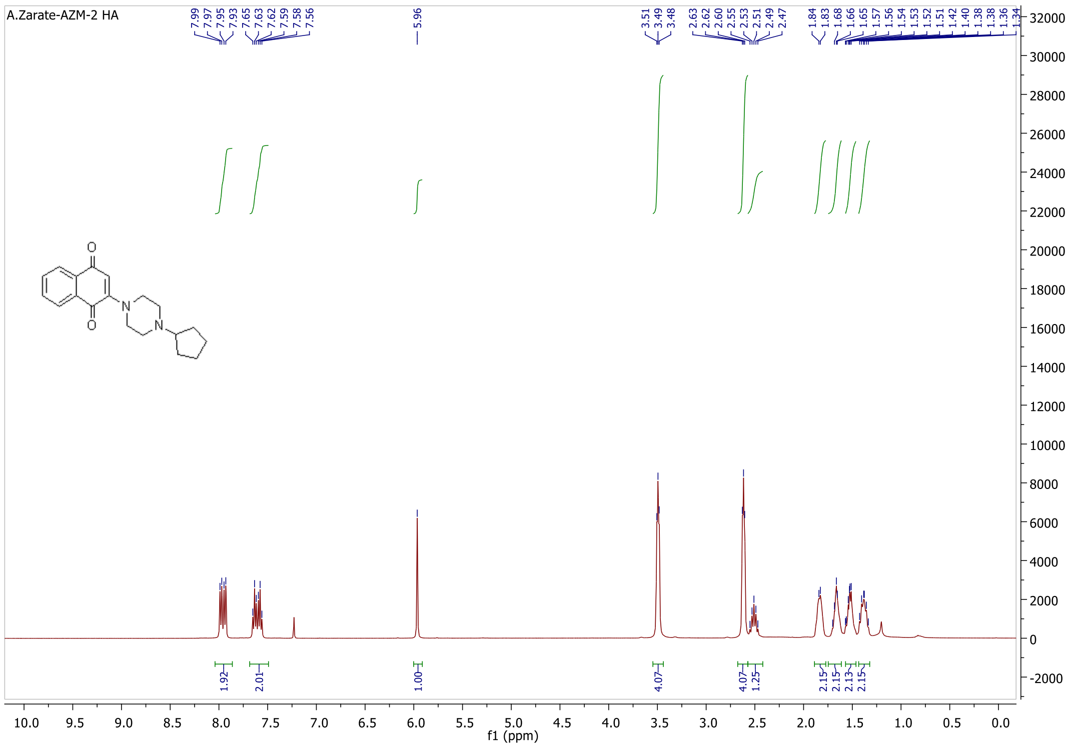

^{13}C NMR for 2d

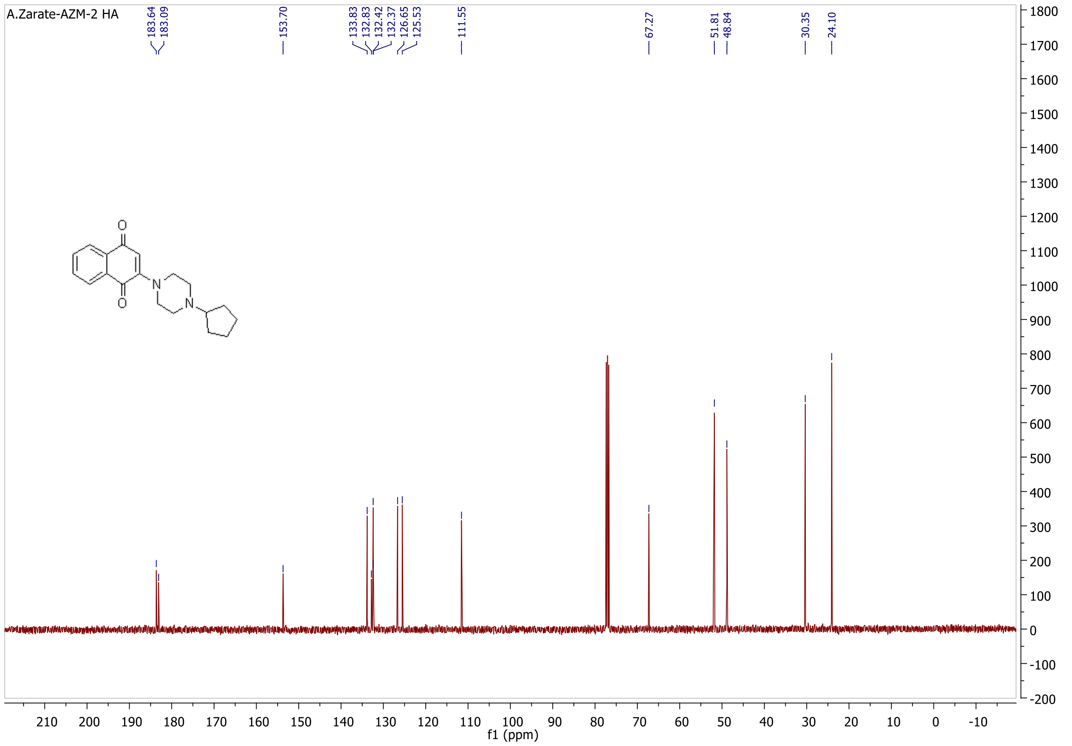

^{1}H NMR for 2e

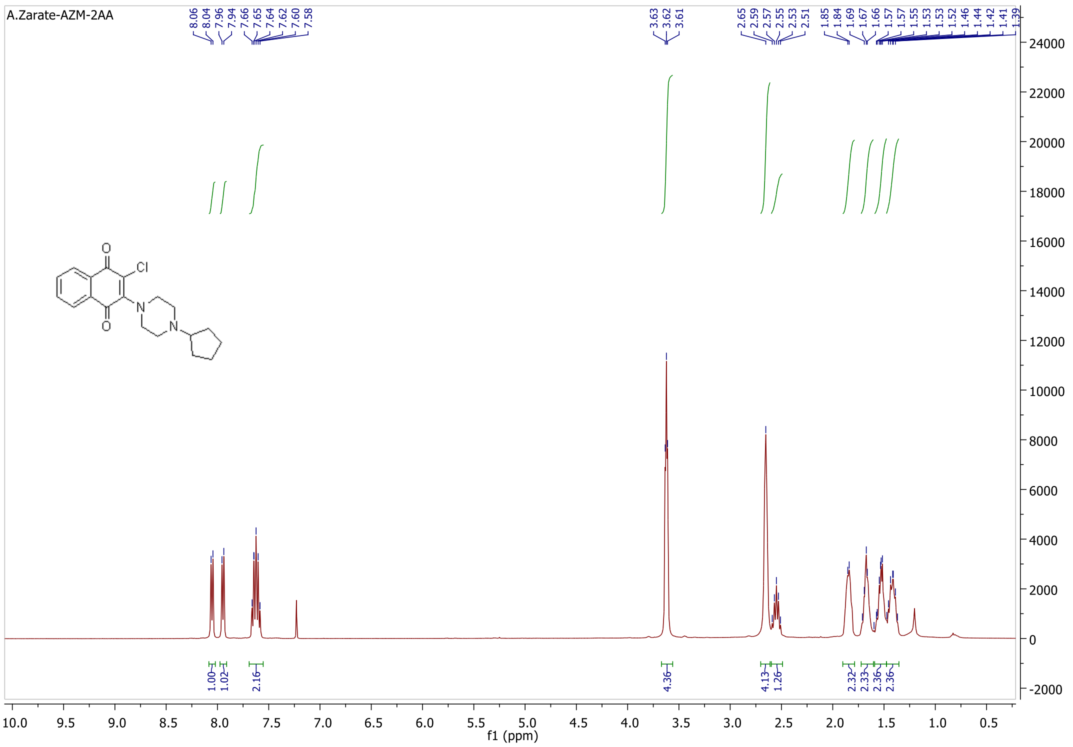

^{13}C NMR for 2e

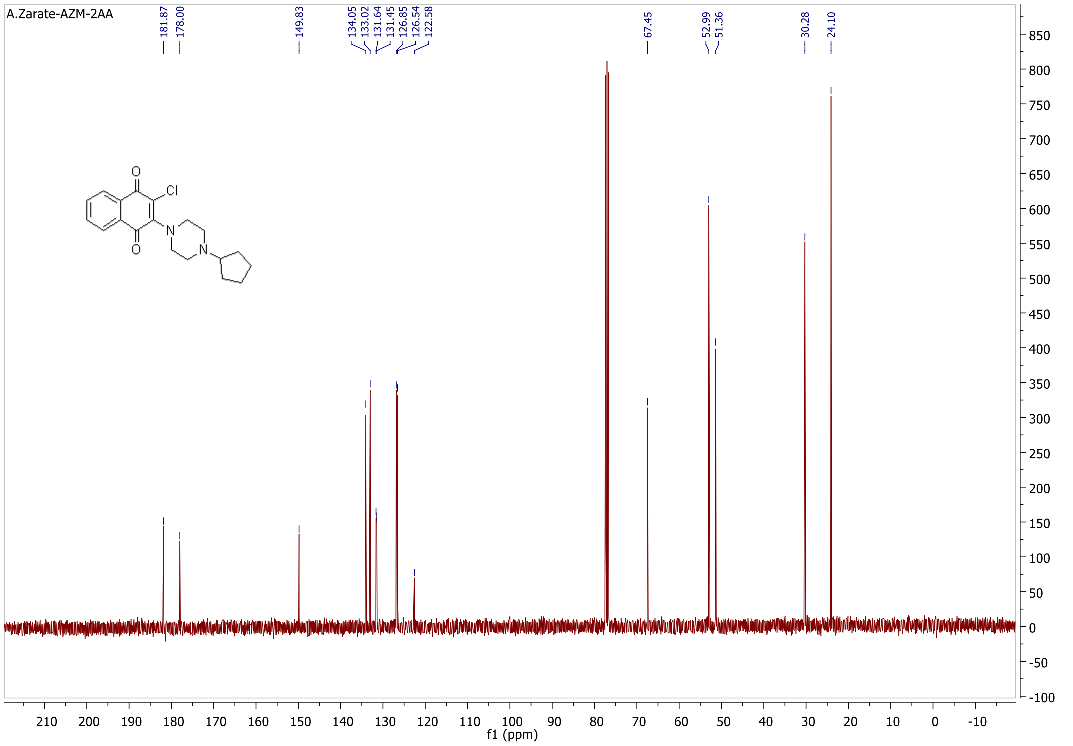

^{1}H NMR for 2f

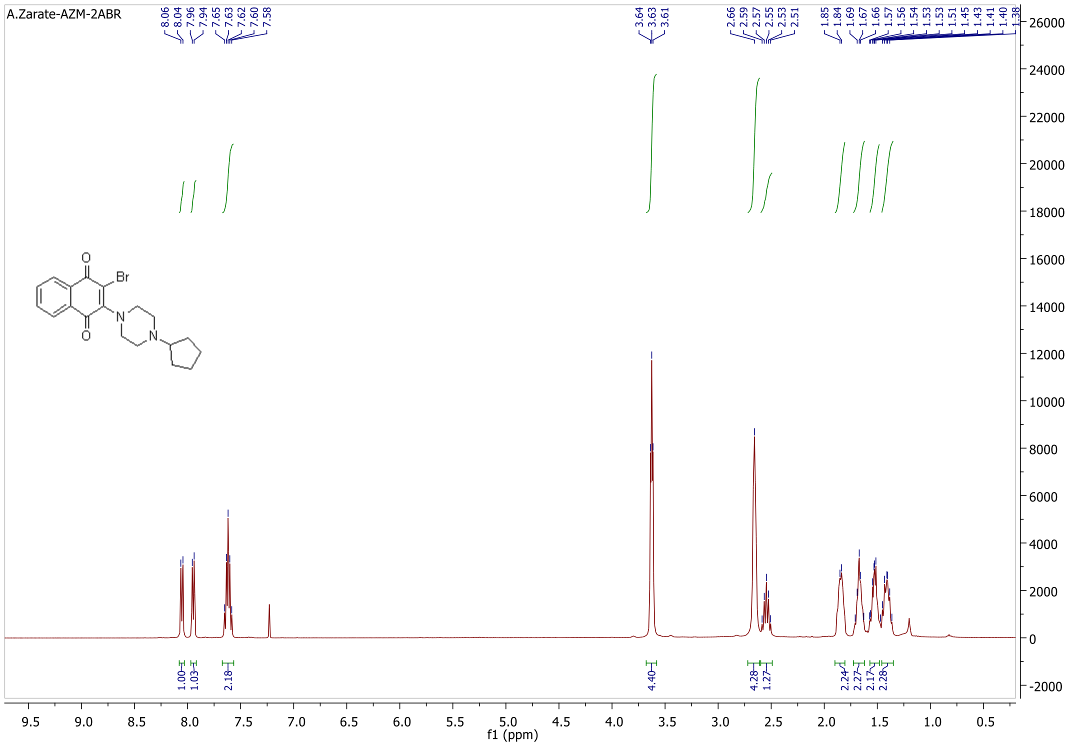

^{13}C NMR for 2f

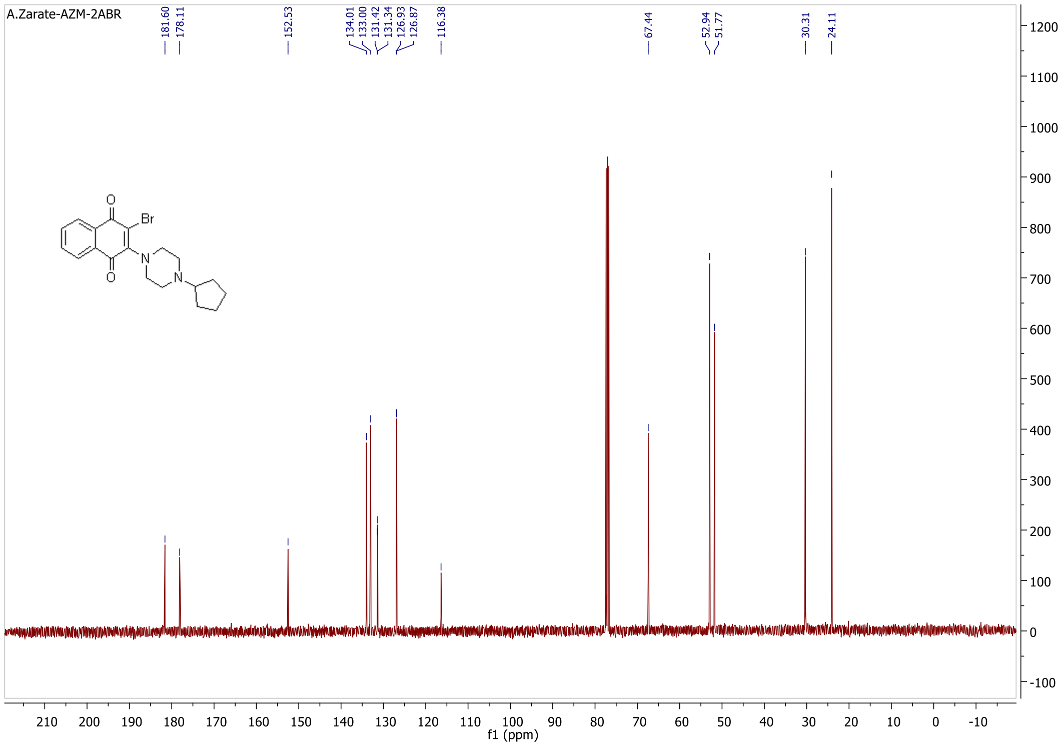

^{1}H NMR for 2g

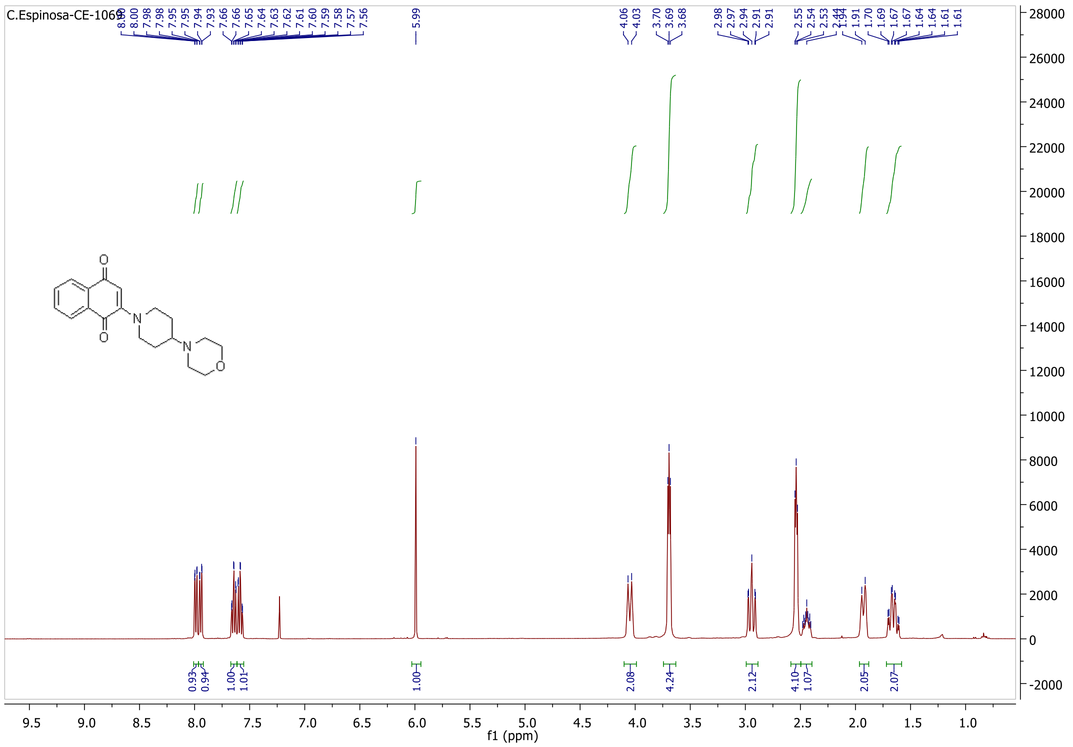

^{13}C NMR for 2g

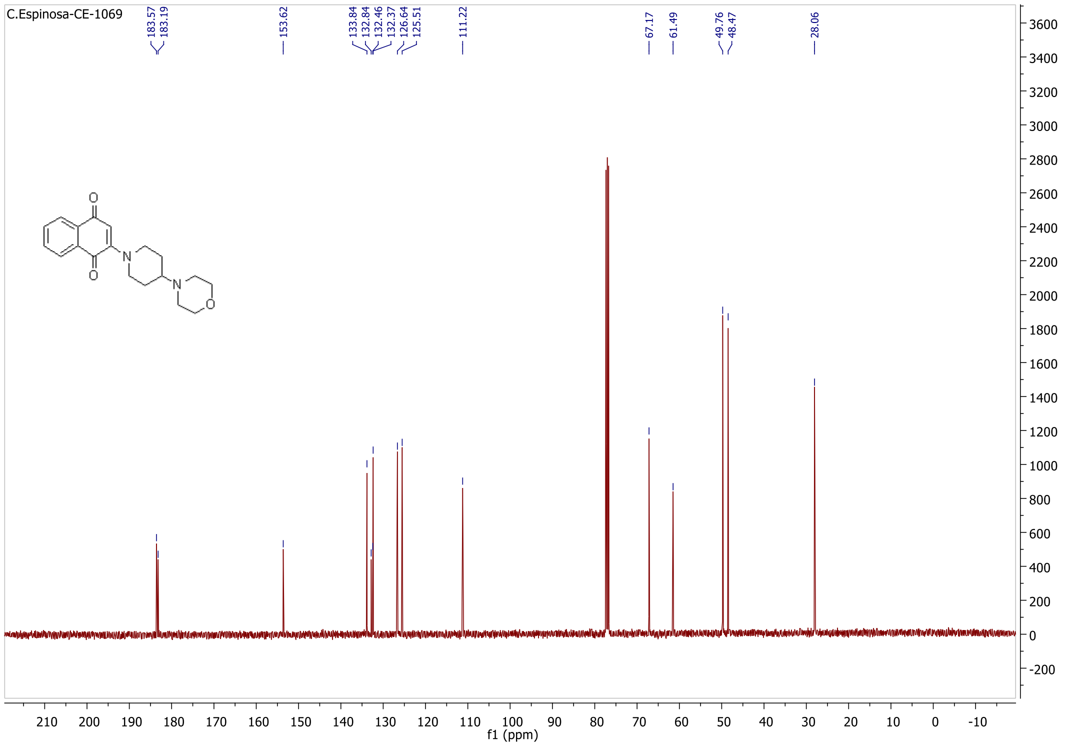

^{1}H NMR for 2h

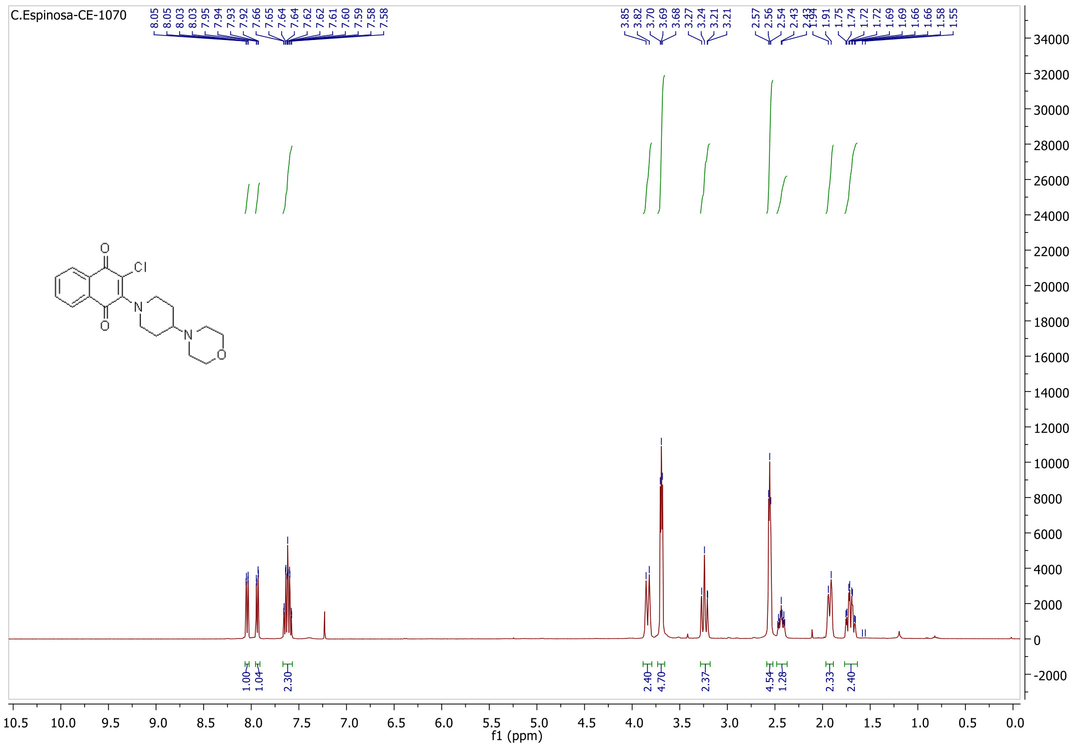

^{13}C NMR for 2h

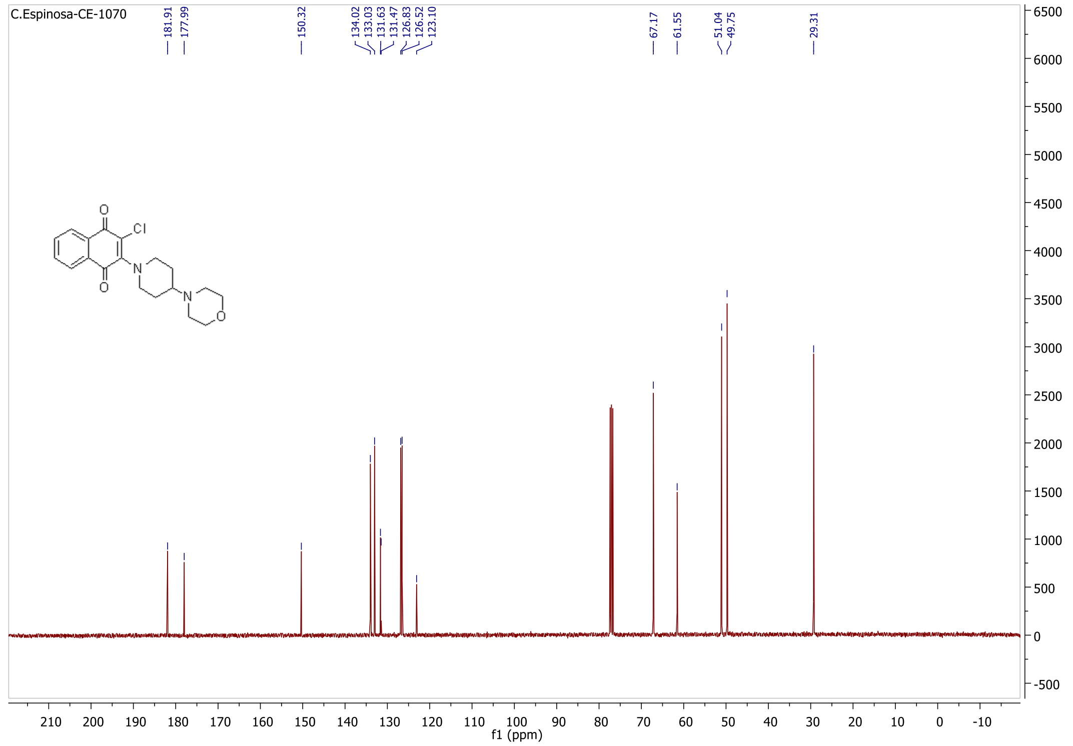

^{1}H NMR for 2i

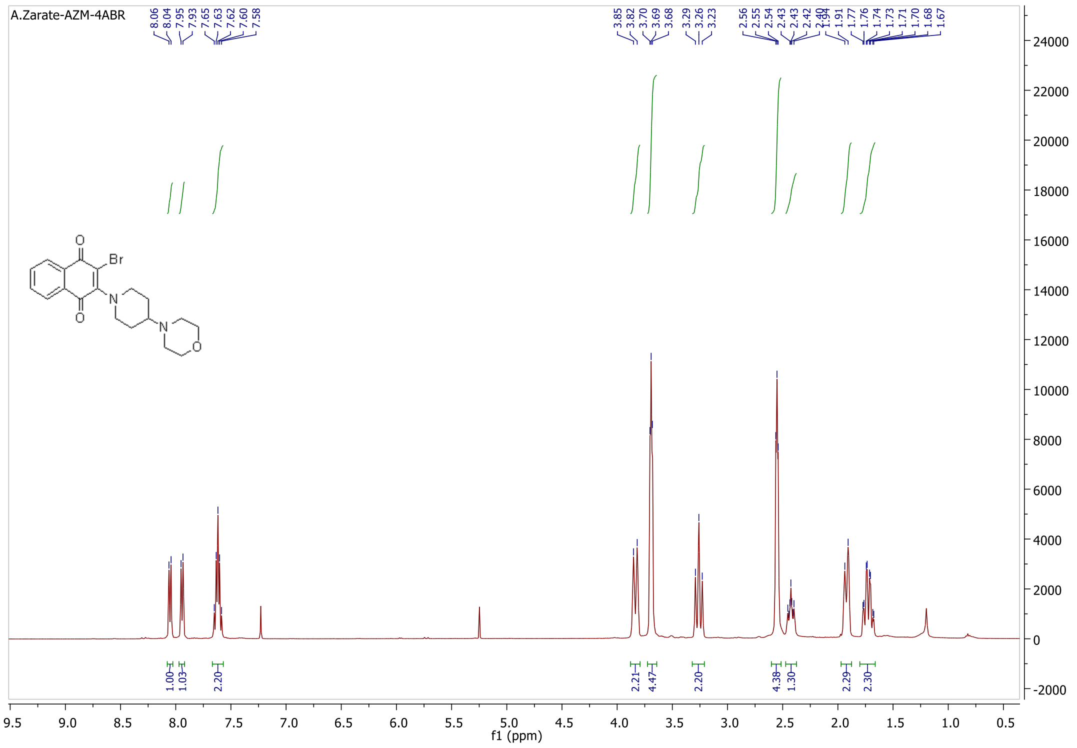

^{13}C NMR for 2i

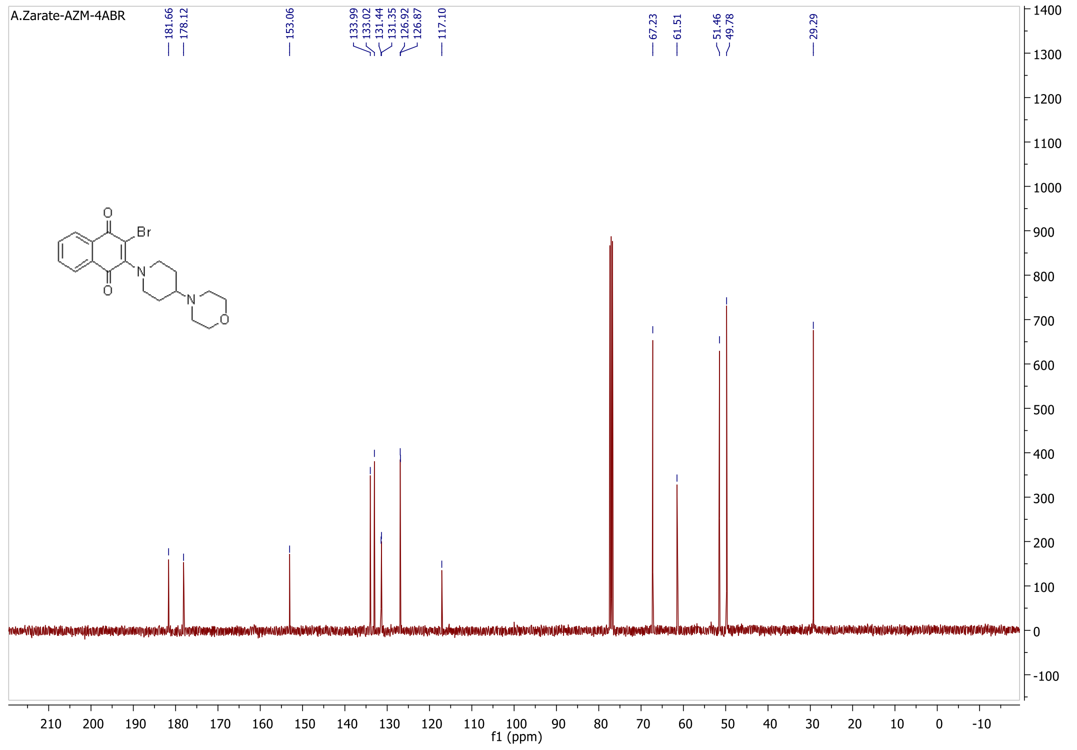

^{1}H NMR for 7a

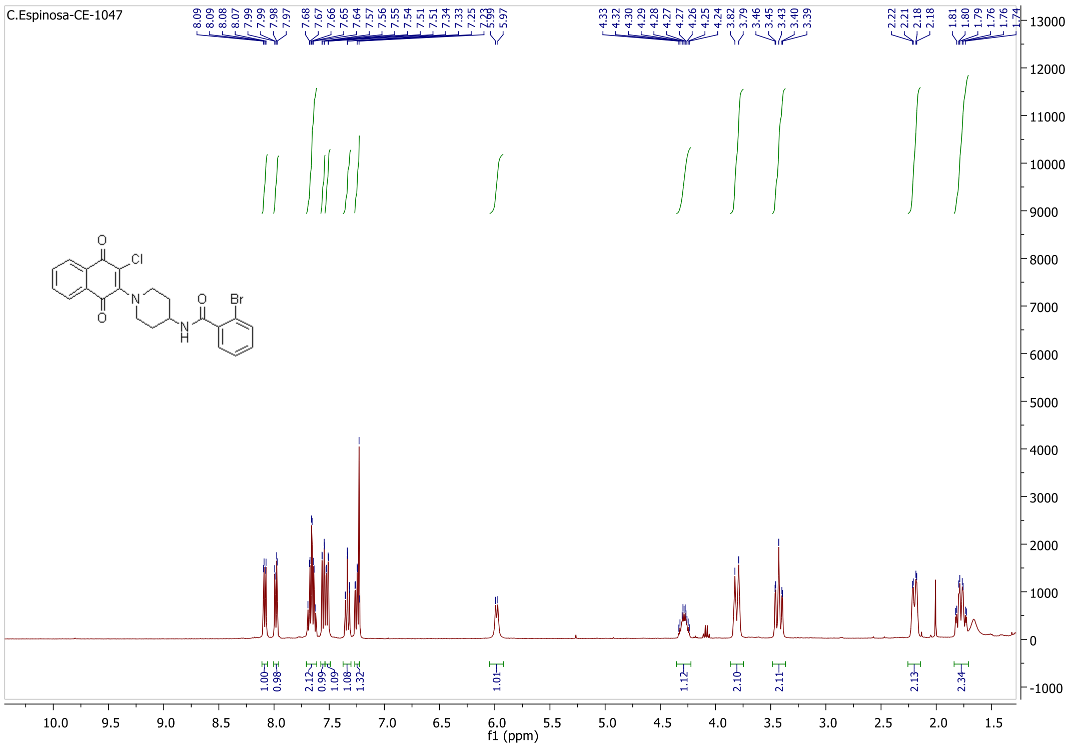

^{13}C NMR for 7a

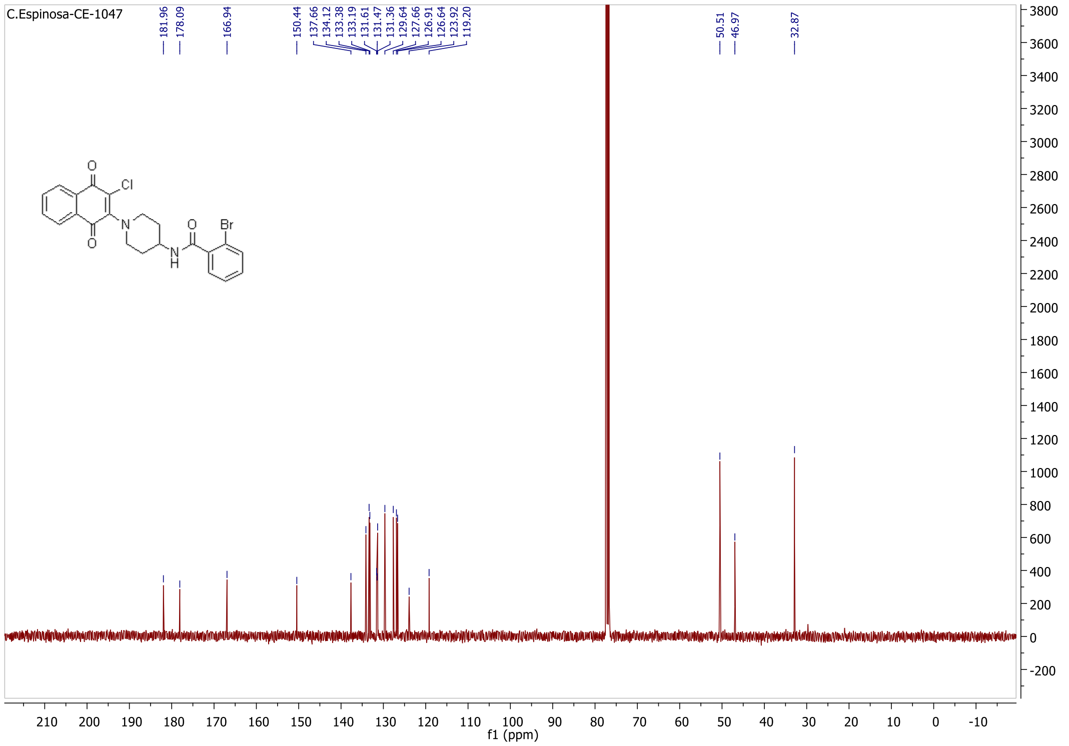

^{1}H NMR for 7b

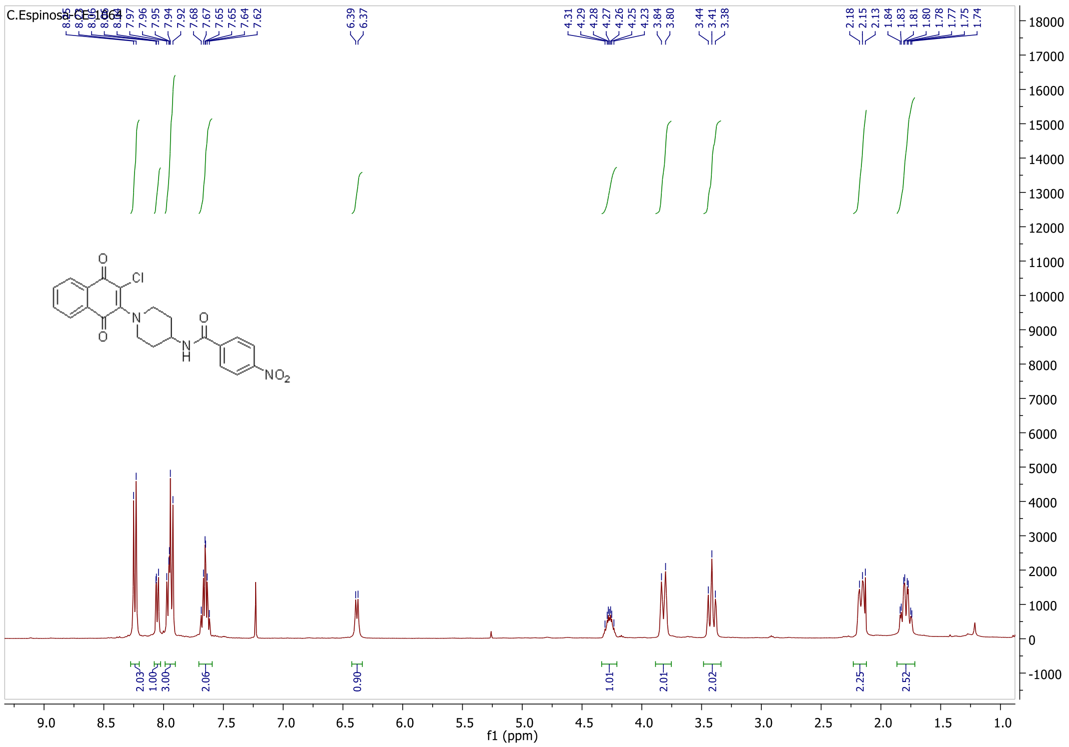

^{13}C NMR for 7b

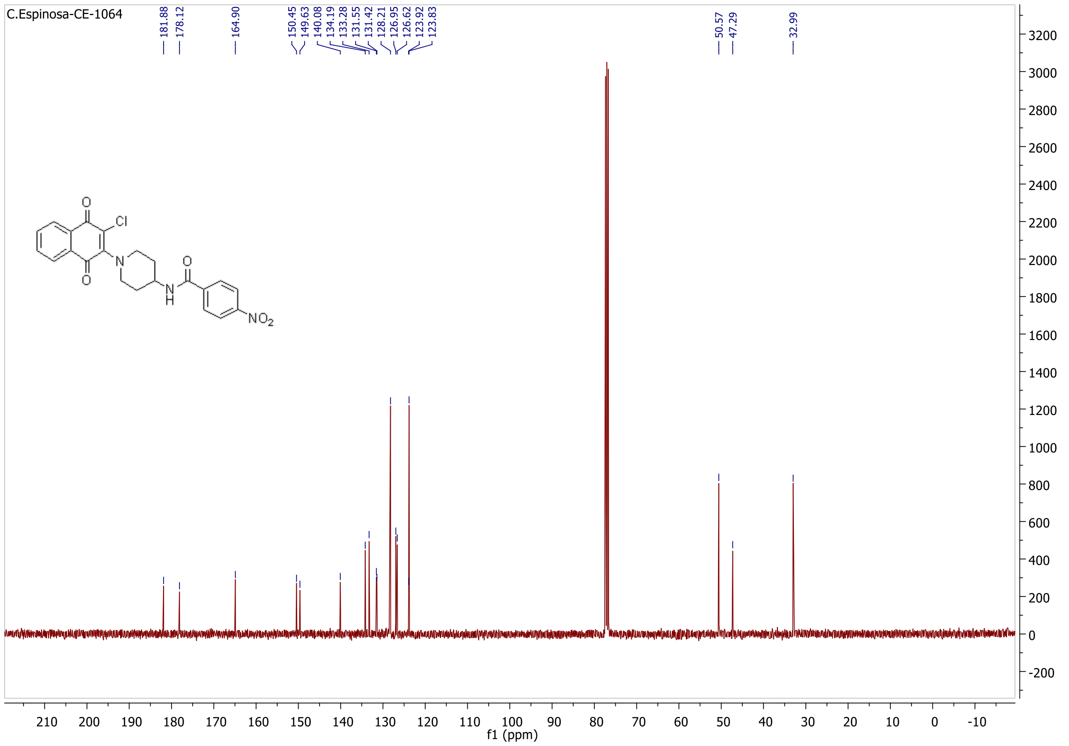

^{1}H NMR for 7c

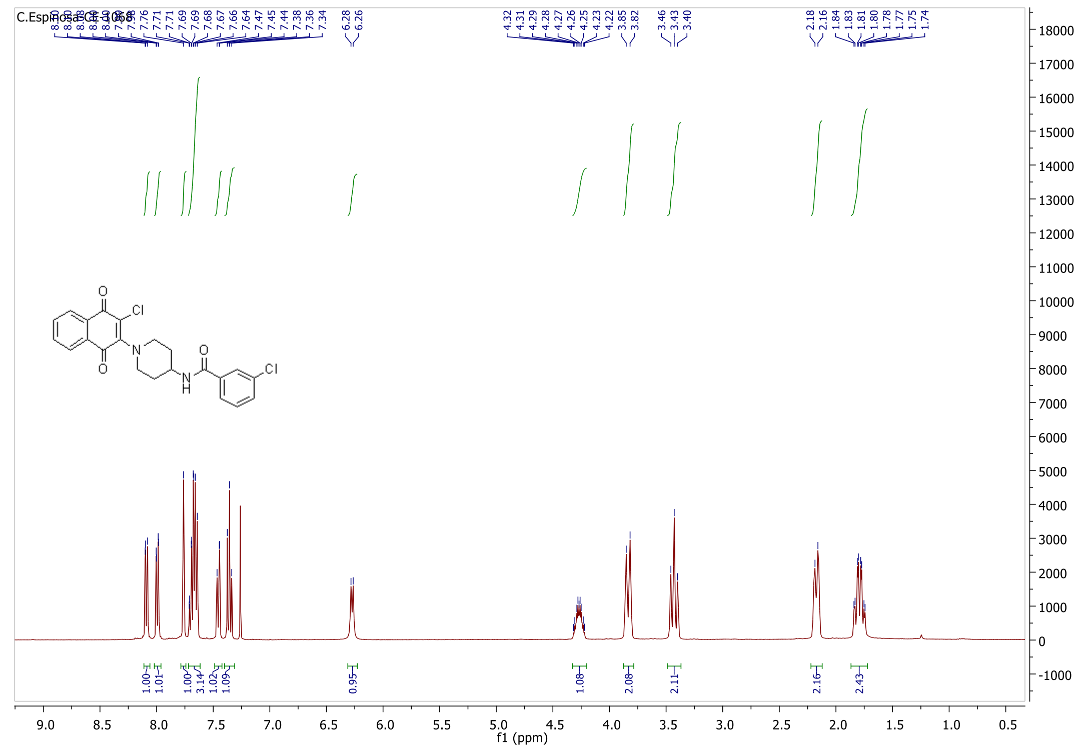

^{13}C NMR for 7c

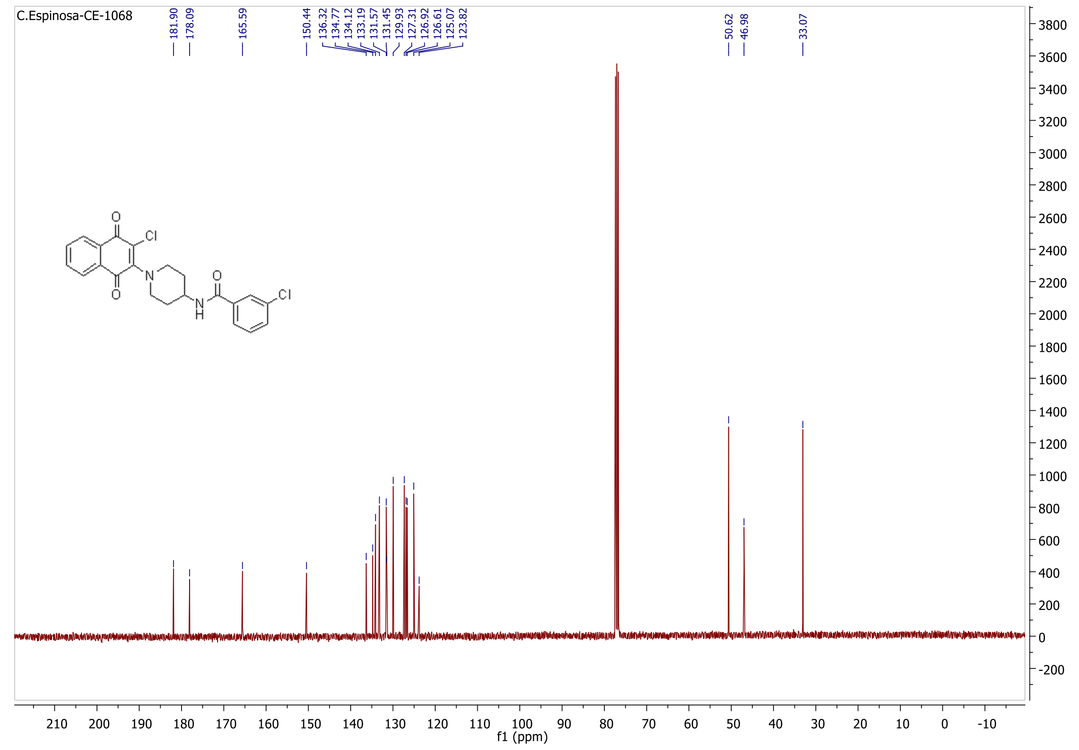

^{1}H NMR for 7d

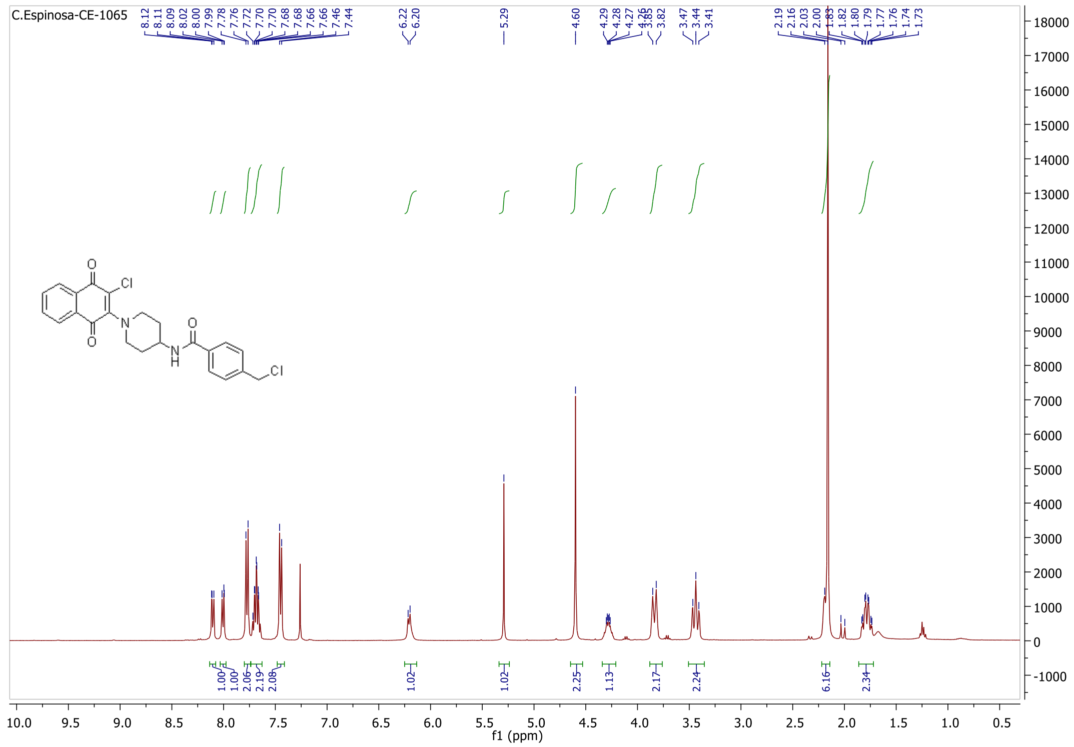

^{13}C NMR for 7d

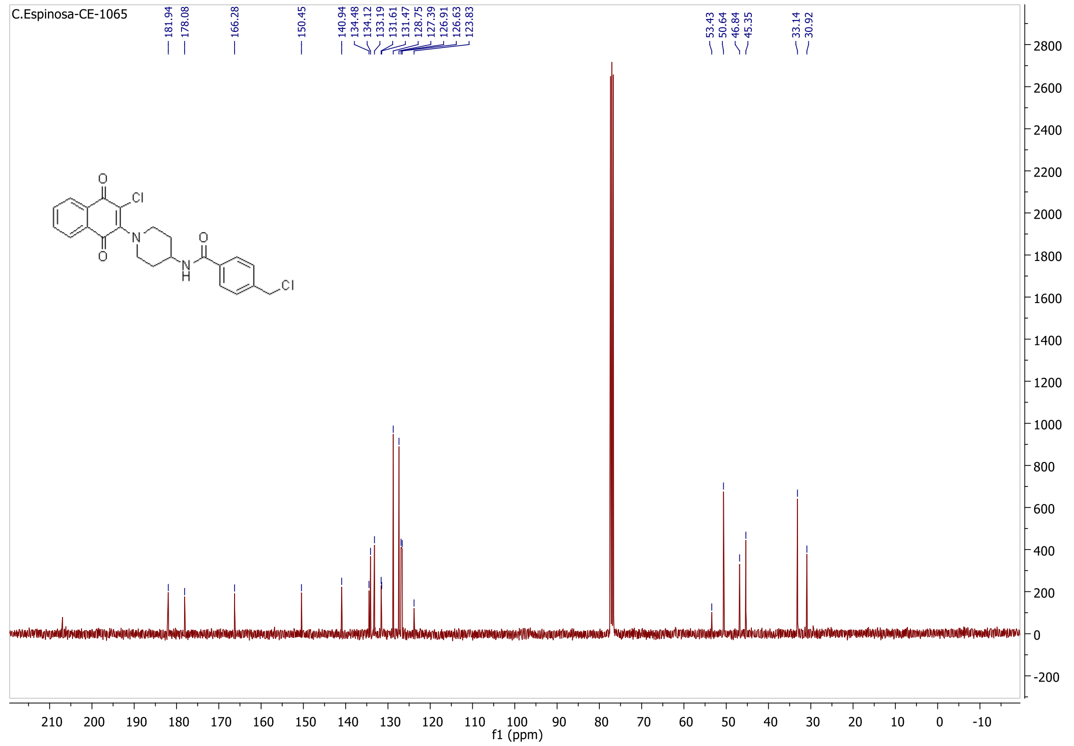

^{1}H NMR for 7e

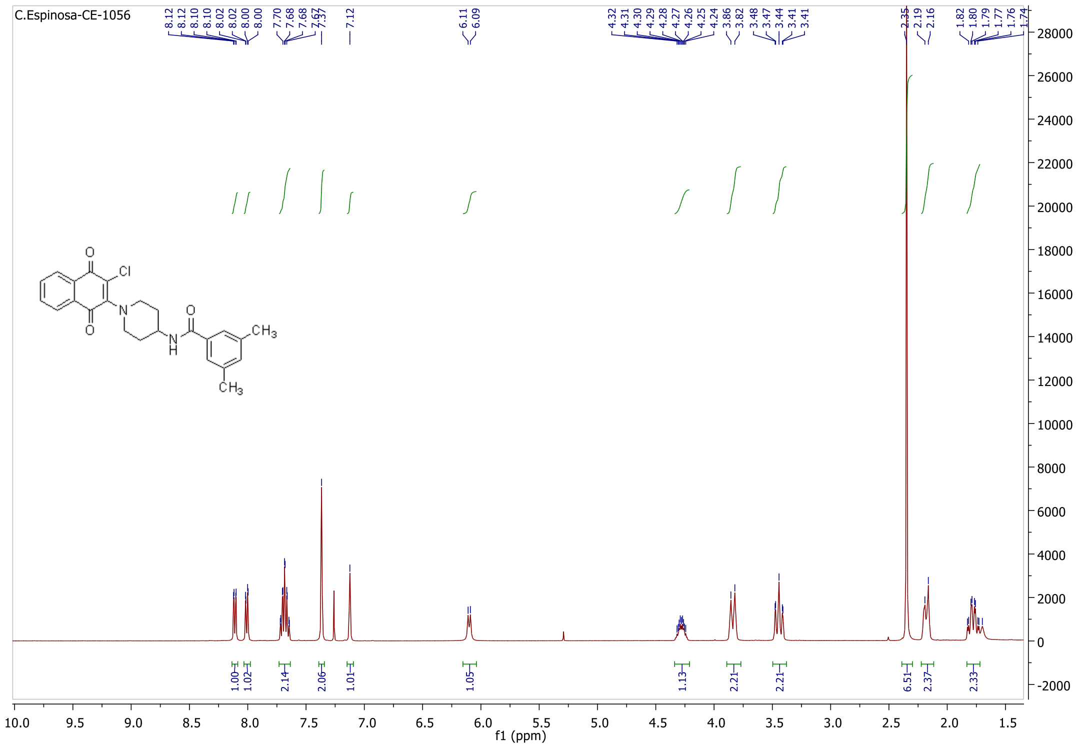

^{13}C NMR for 7e

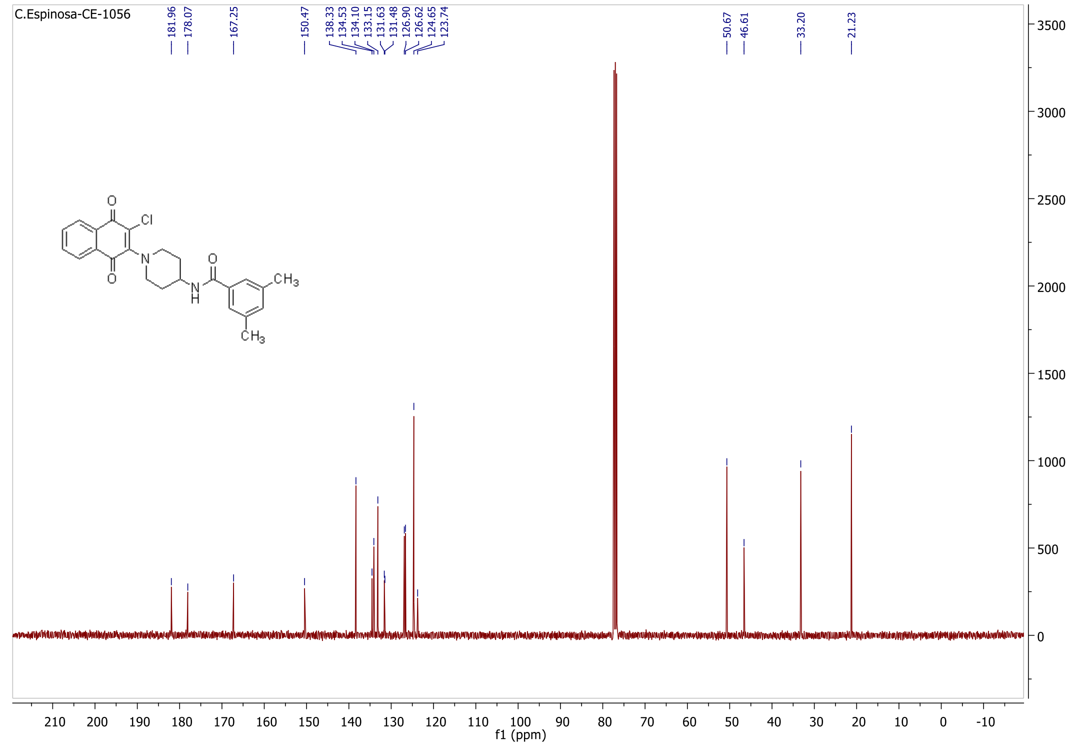

^{1}H NMR for 7g

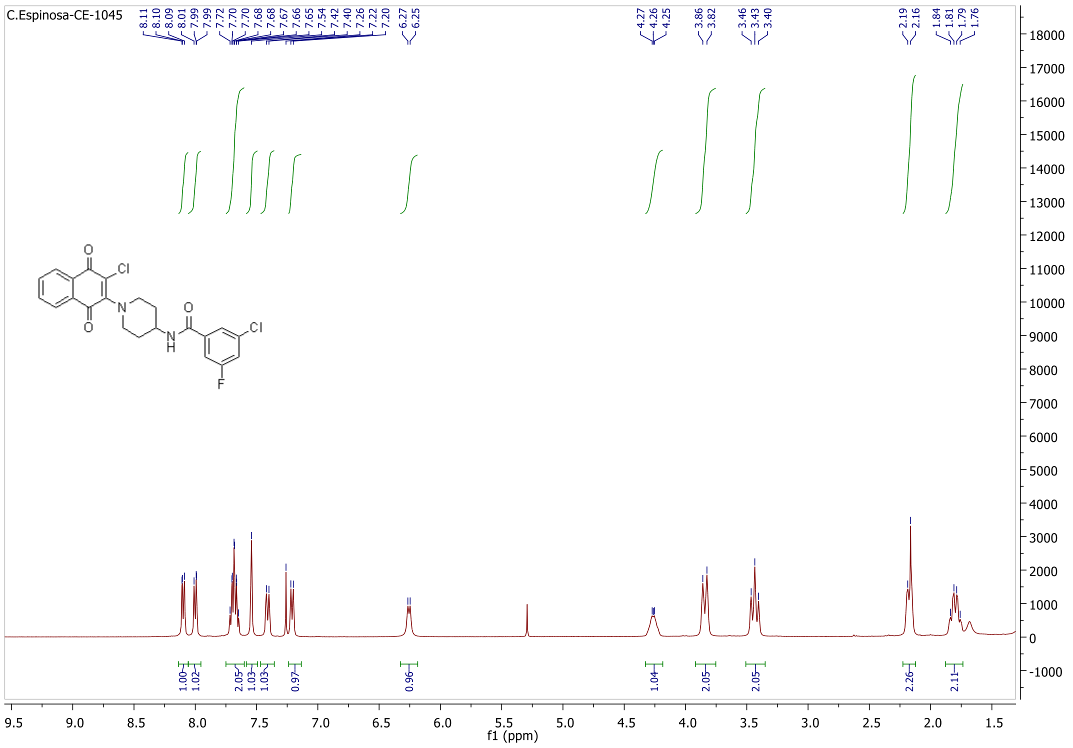

^{13}C NMR for 7g

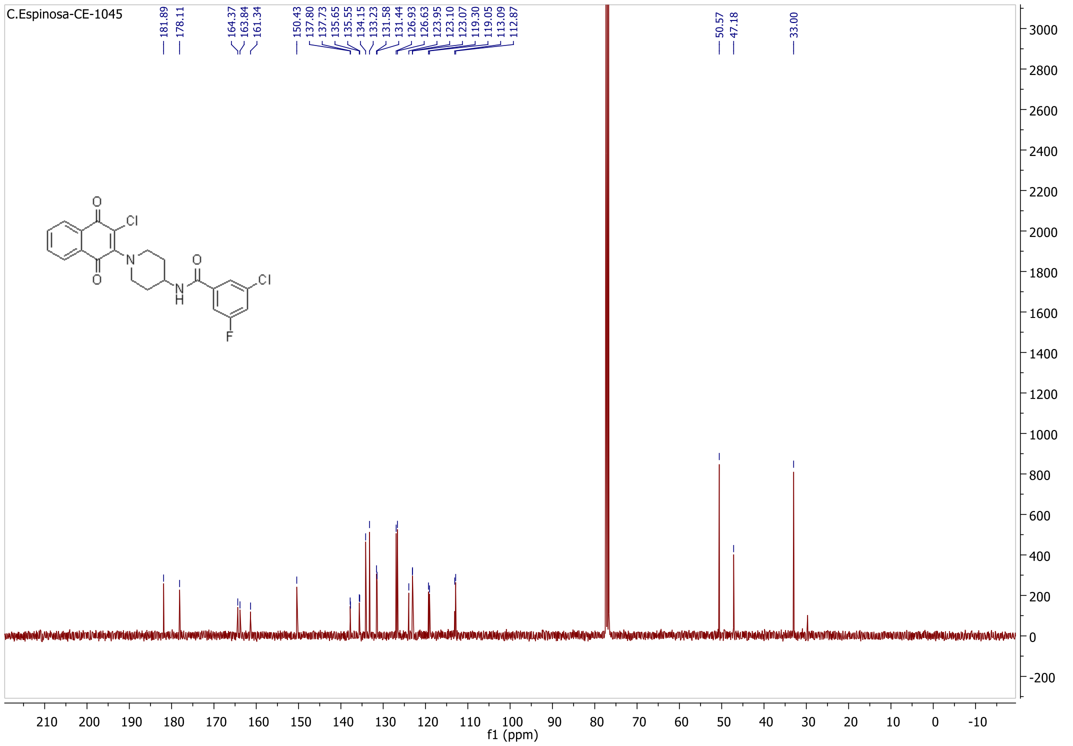

^{1}H NMR for 7h

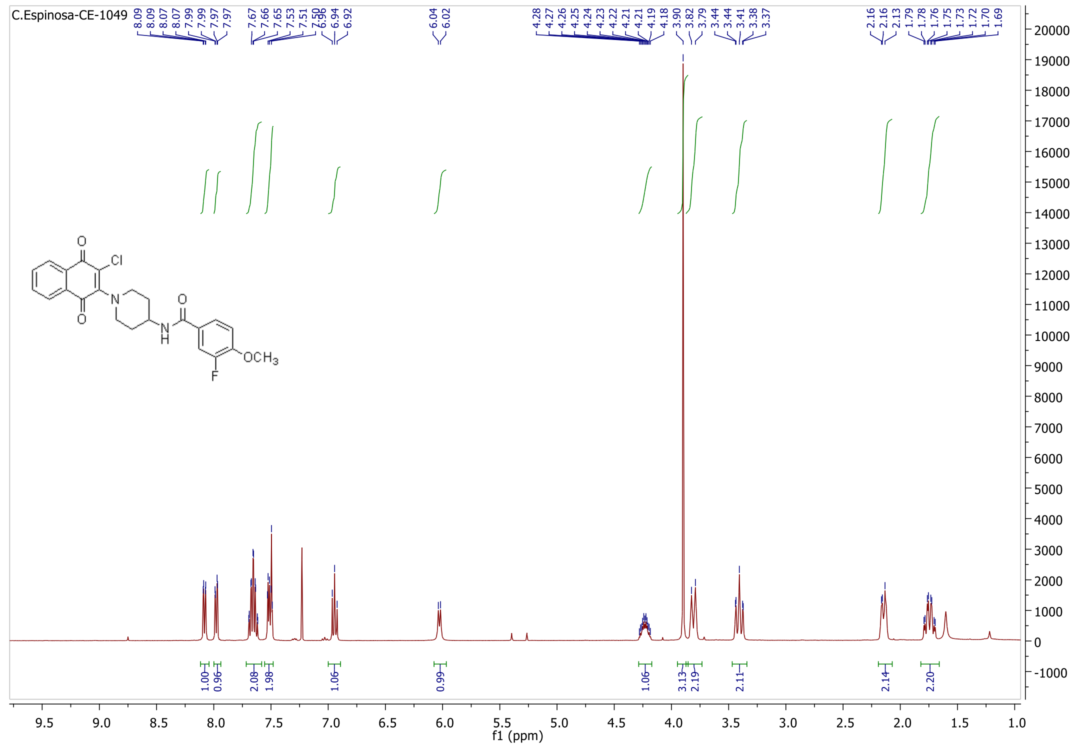

^{13}C NMR for 7h

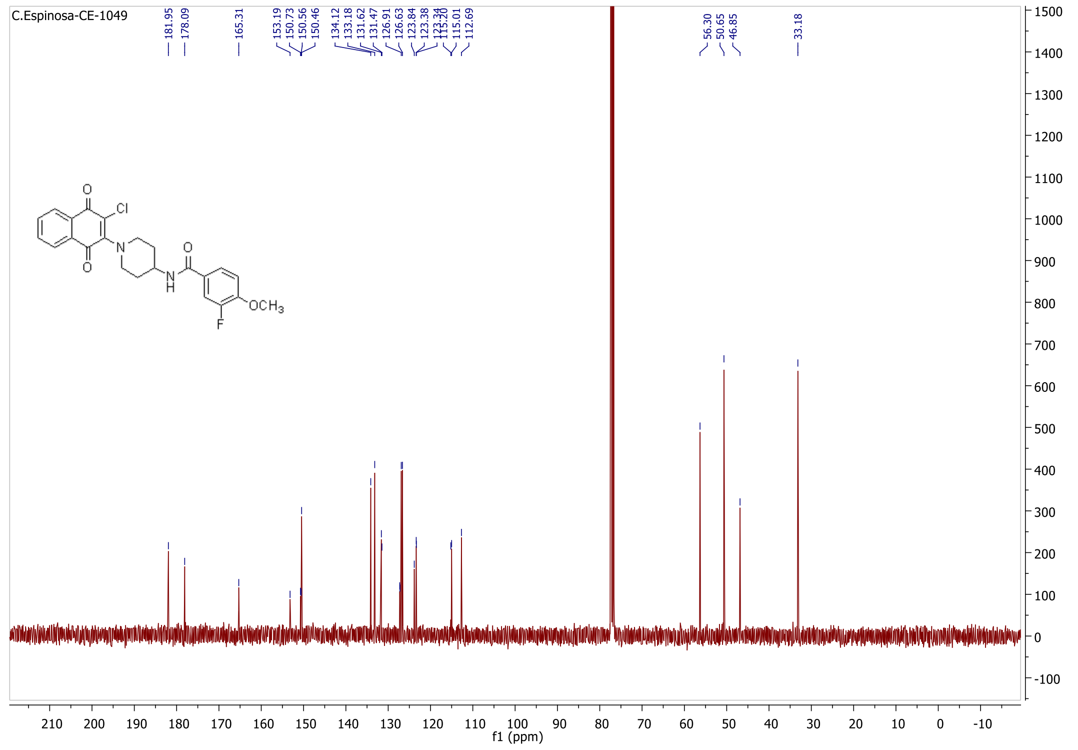

^{1}H NMR for 7i

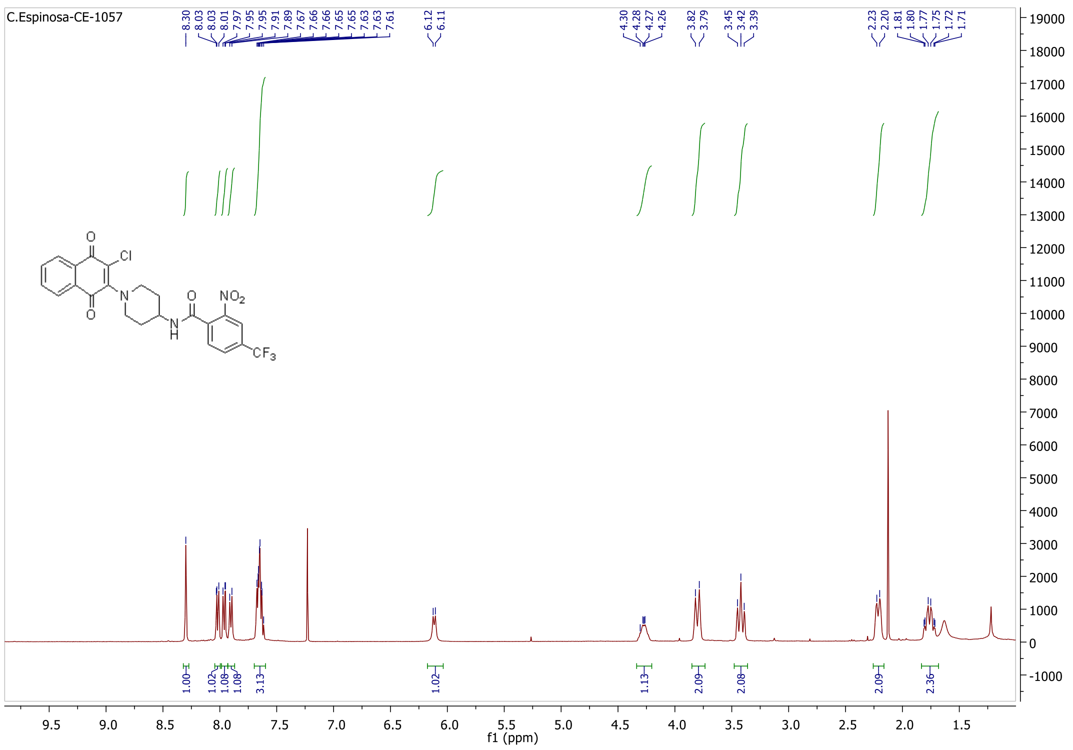

^{13}C NMR for 7i

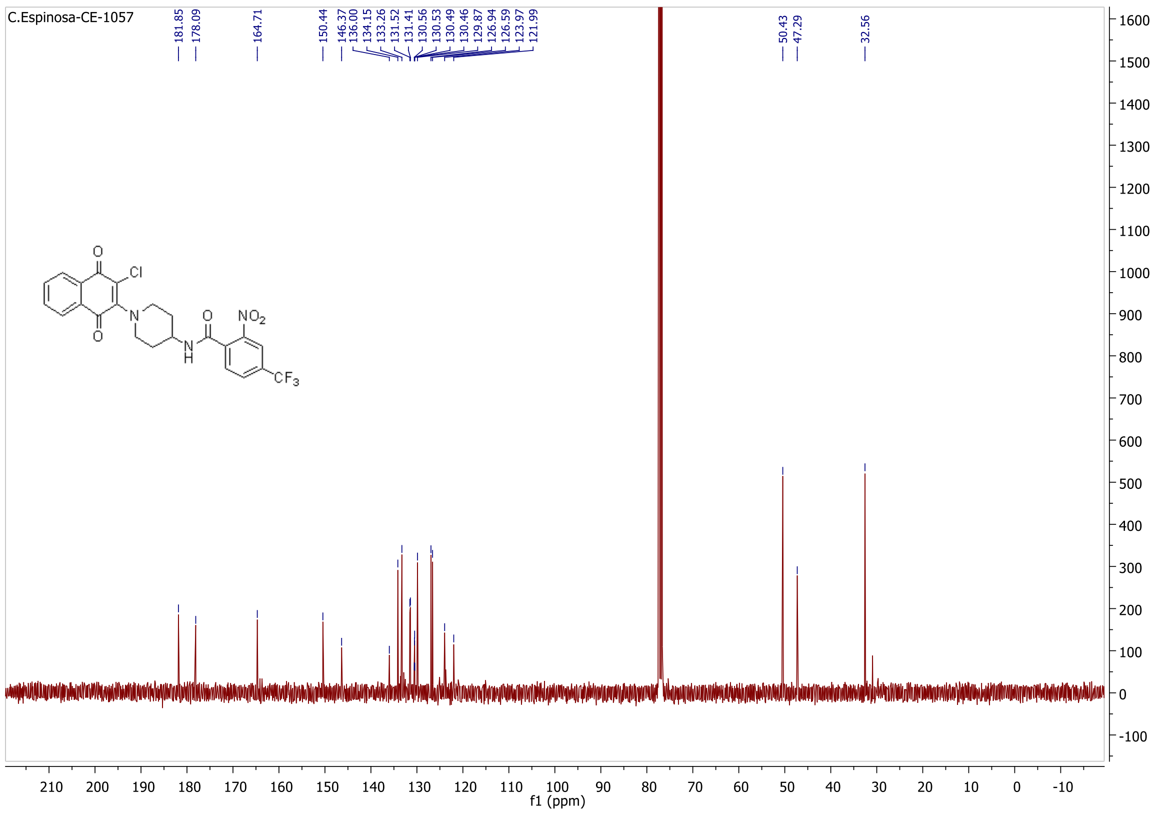

^{1}H NMR for 7j

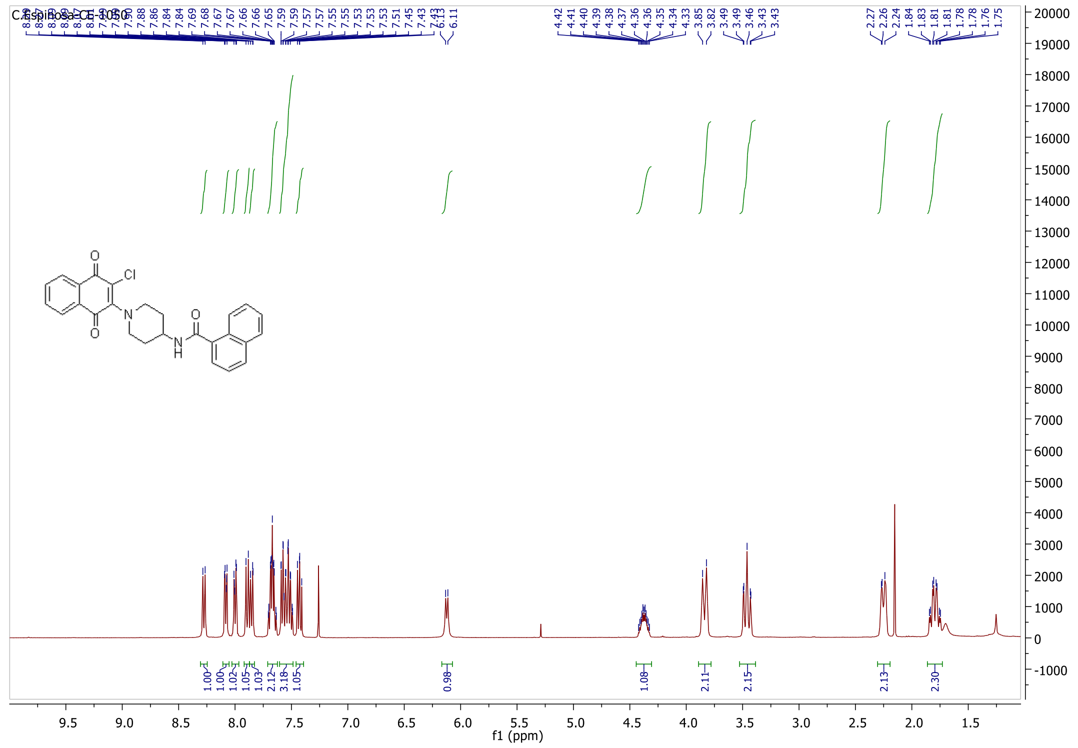

^{13}C NMR for 7j

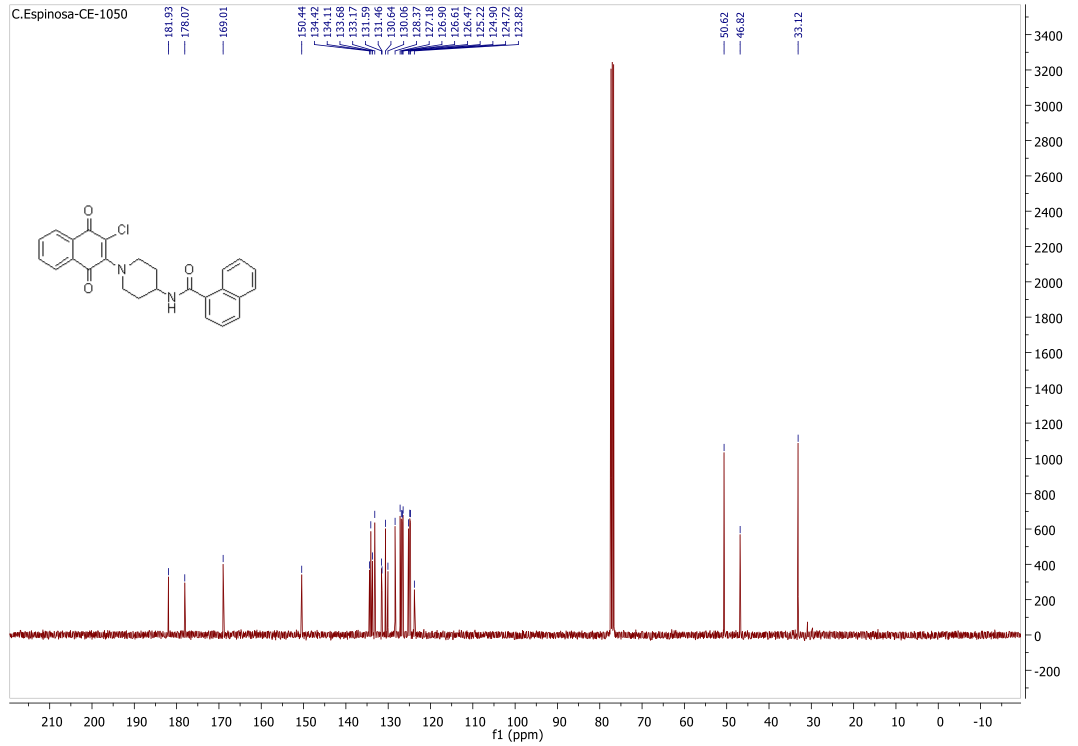

Mass spectra compound 2c

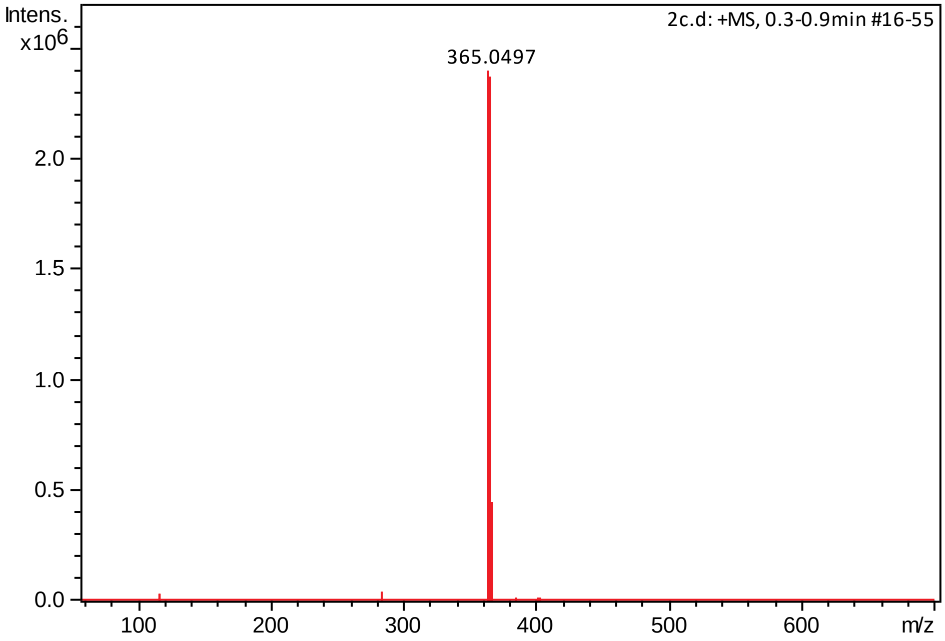

Mass spectra compound 2d

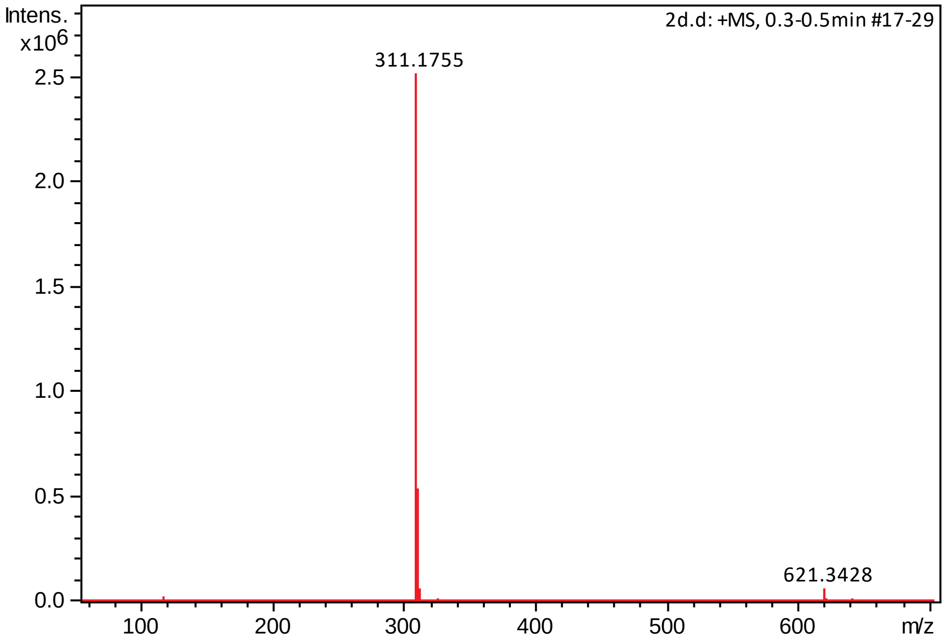

Mass spectra compound 2e

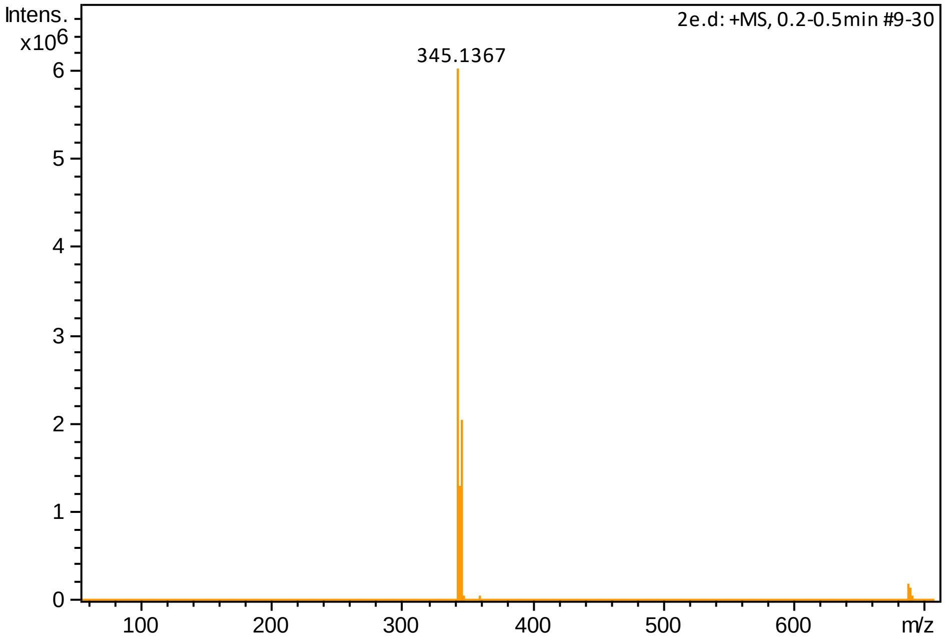

Mass spectra compound 2f

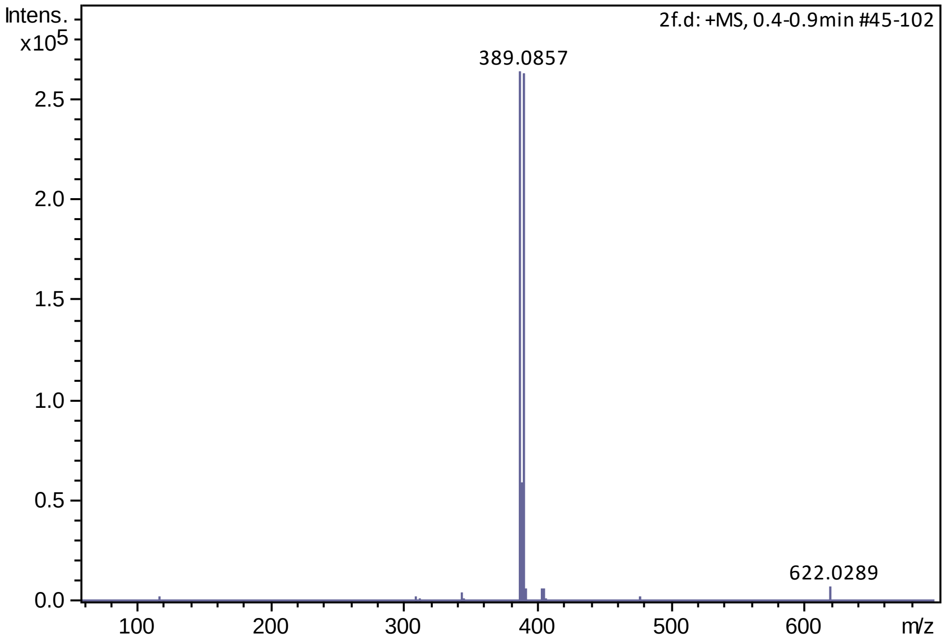

Mass spectra compound 2g

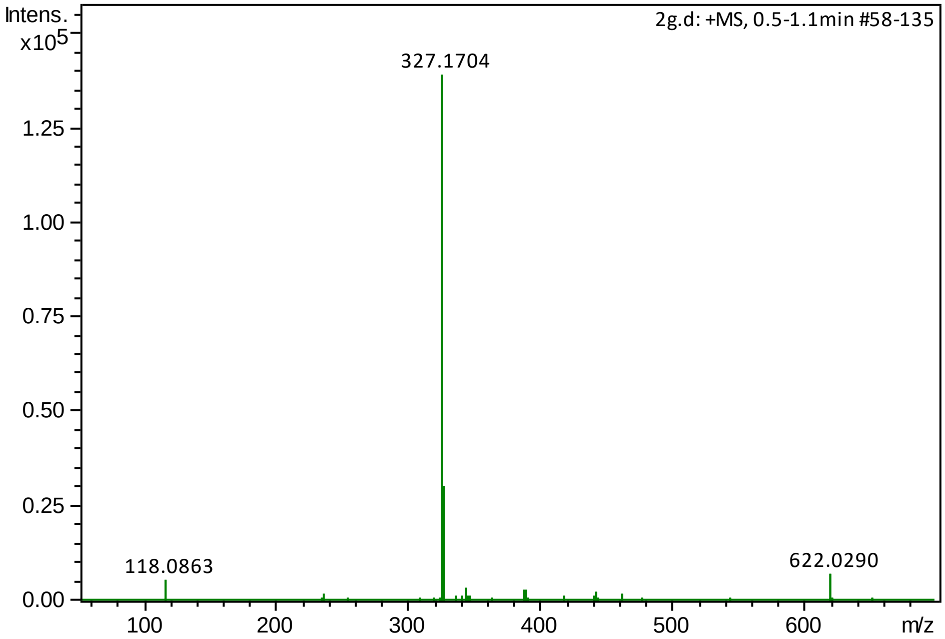

Mass spectra compound 2h

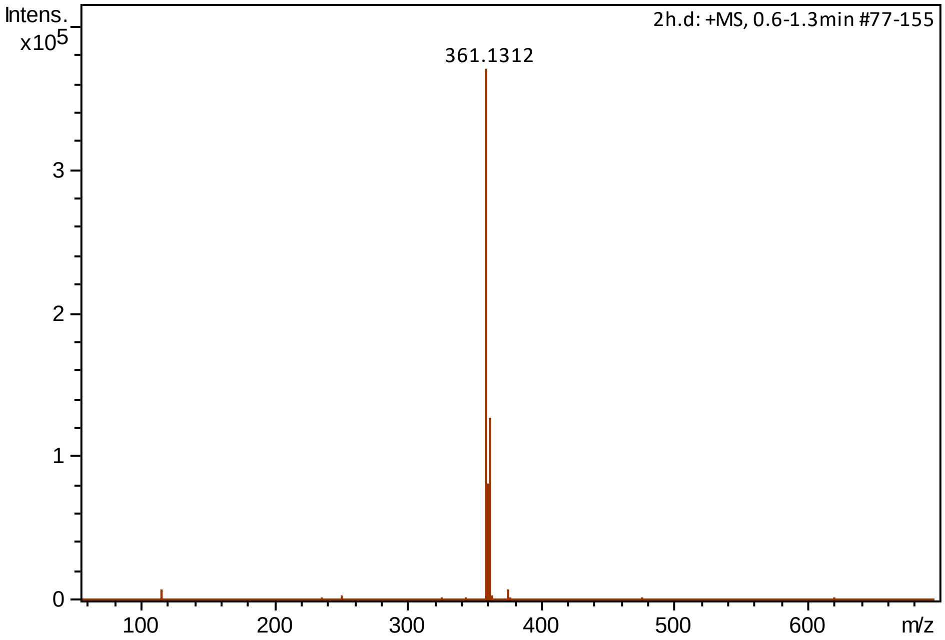

Mass spectra compound 2i

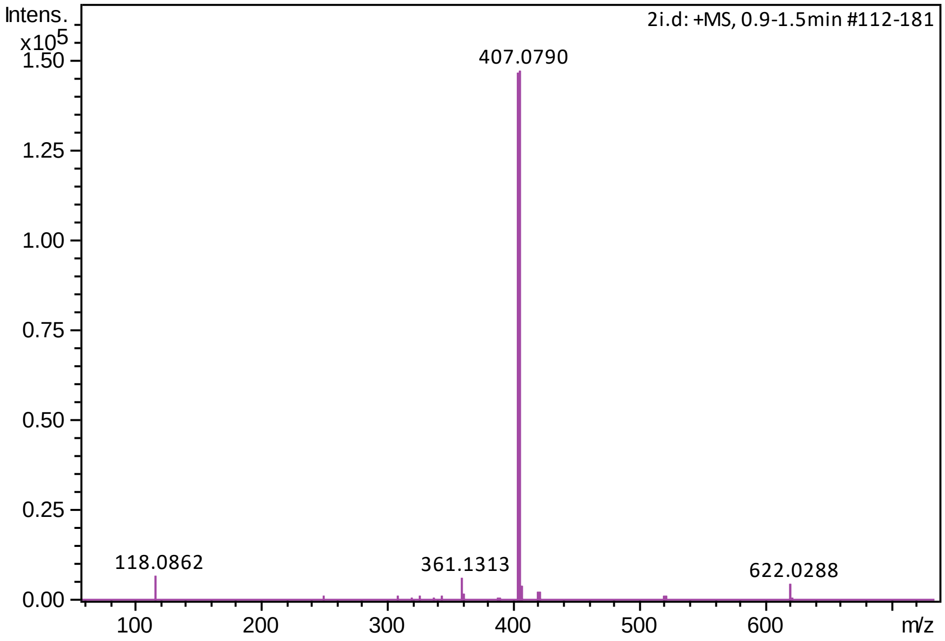

Mass spectra compound 7a

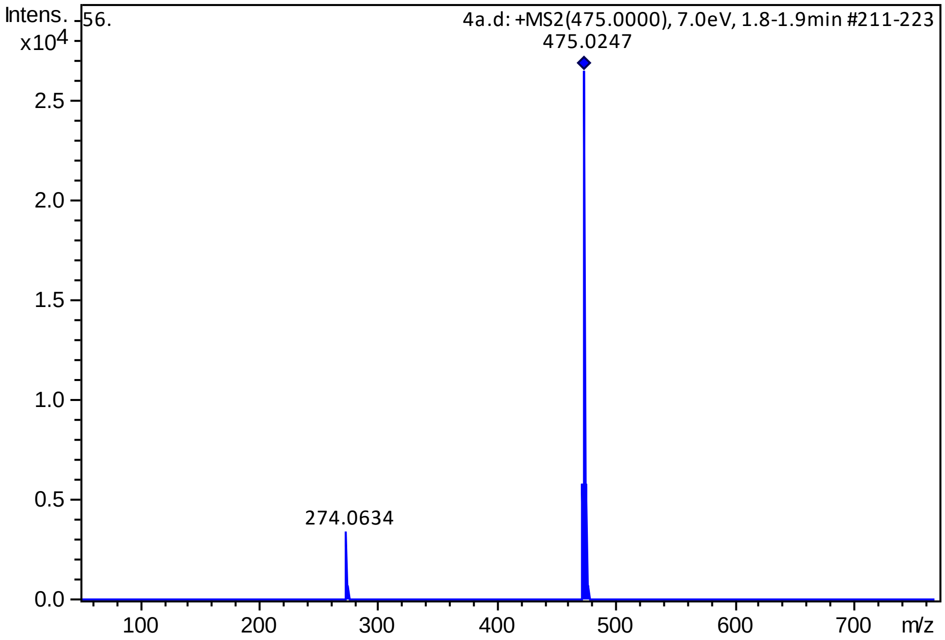

Mass spectra compound 7b

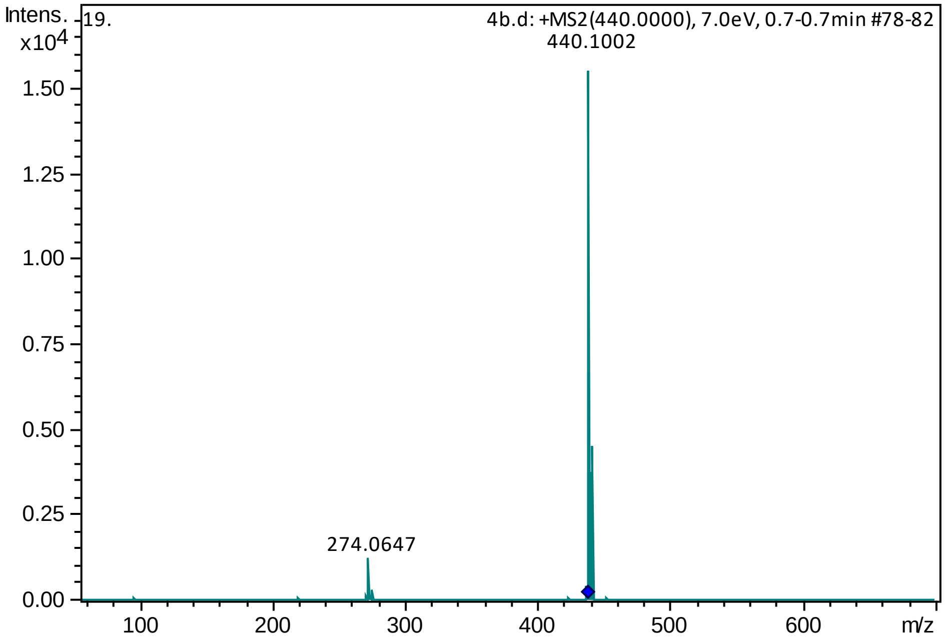

Mass spectra compound 7c

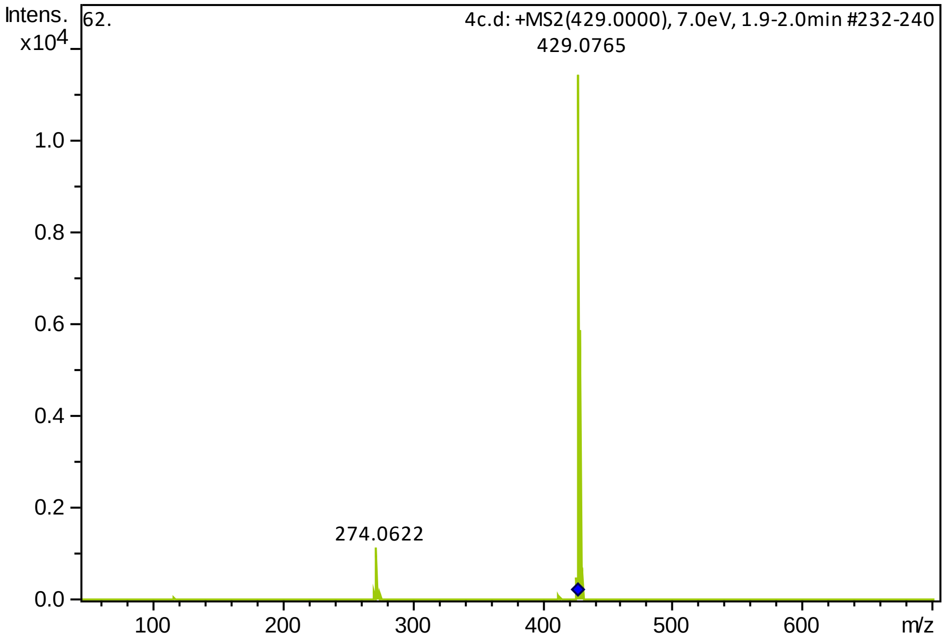

Mass spectra compound 7d

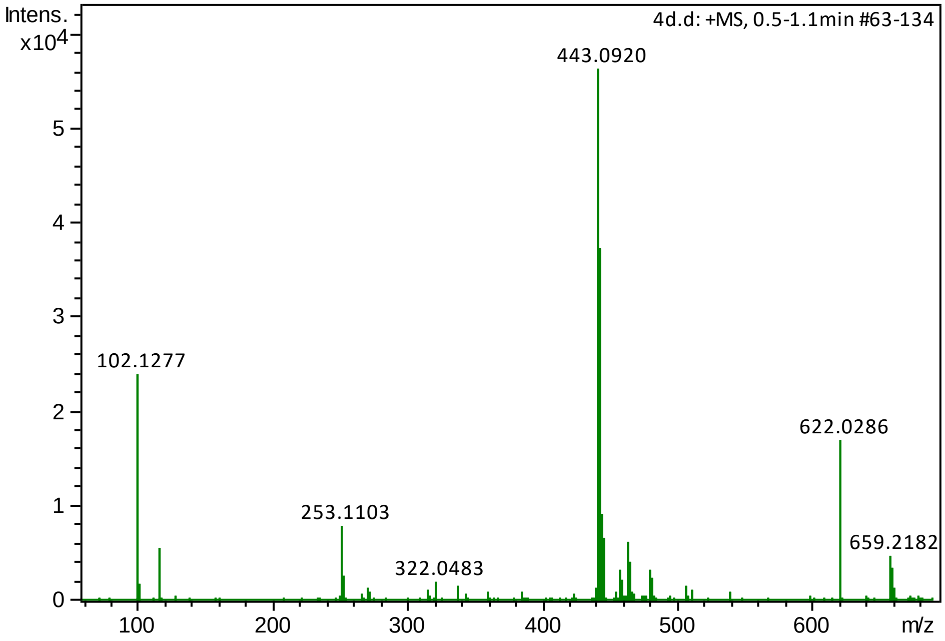

Mass spectra compound 7e

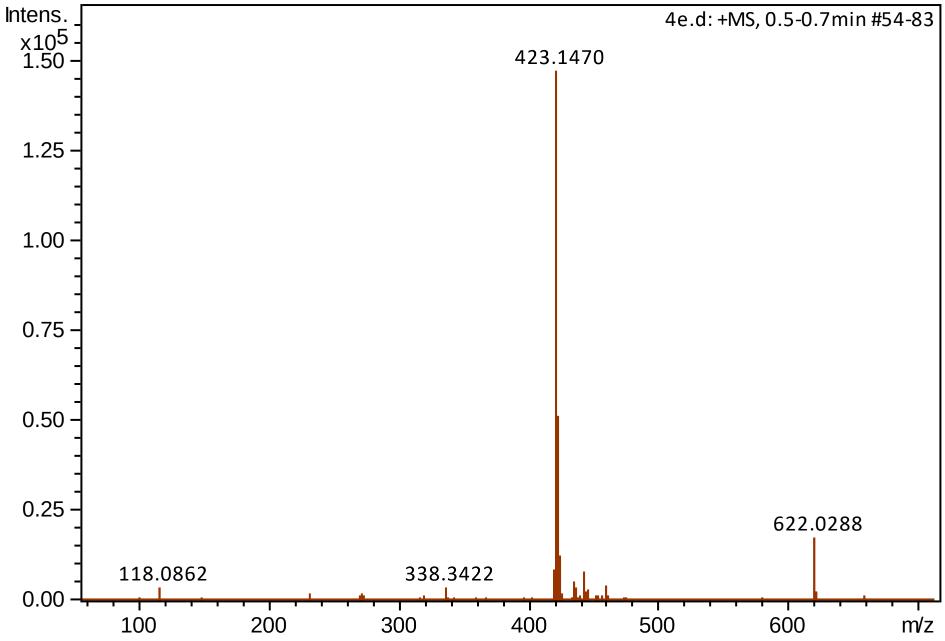

Mass spectra compound 7f

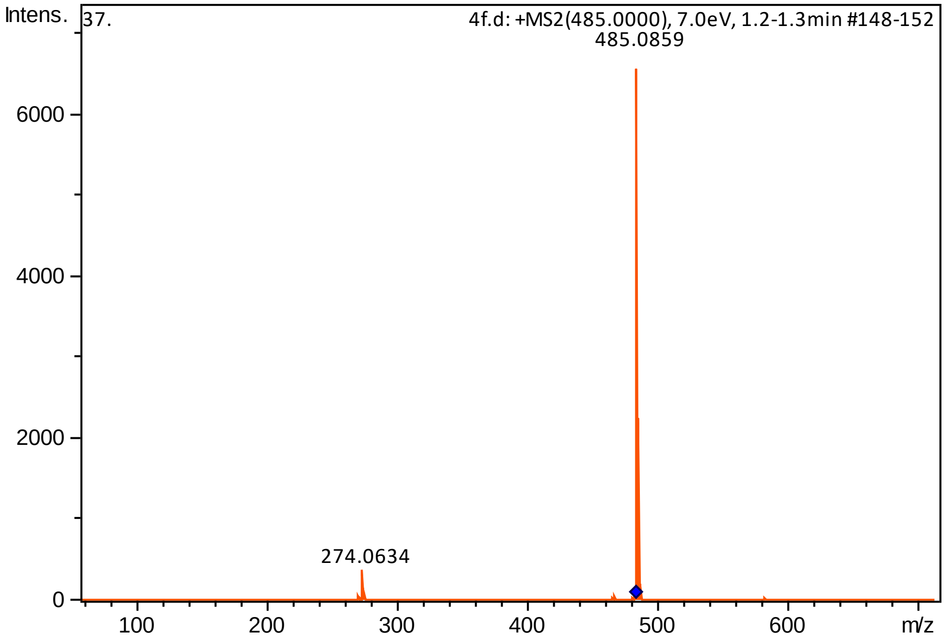

Mass spectra compound 7g

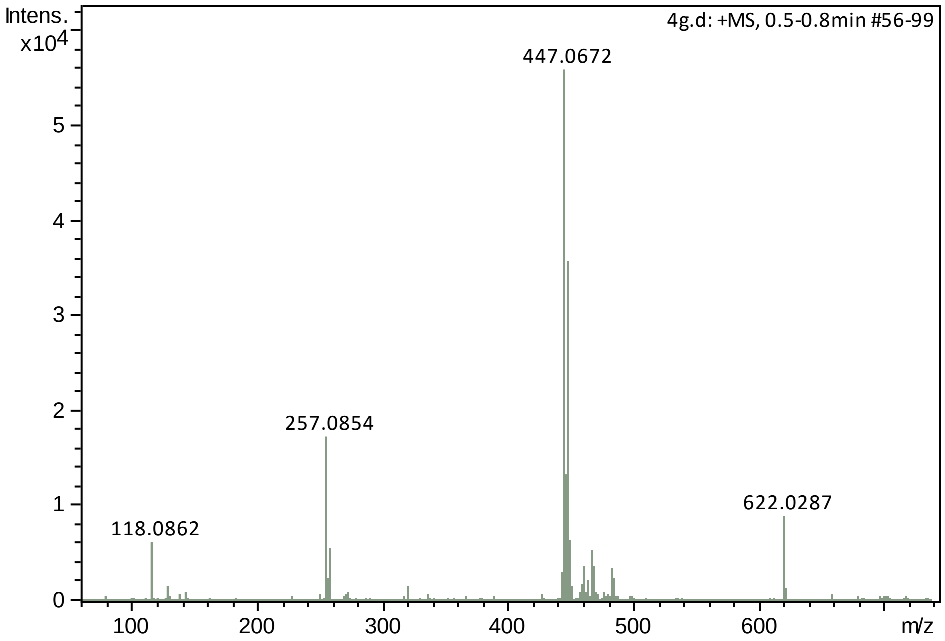

Mass spectra compound 7h

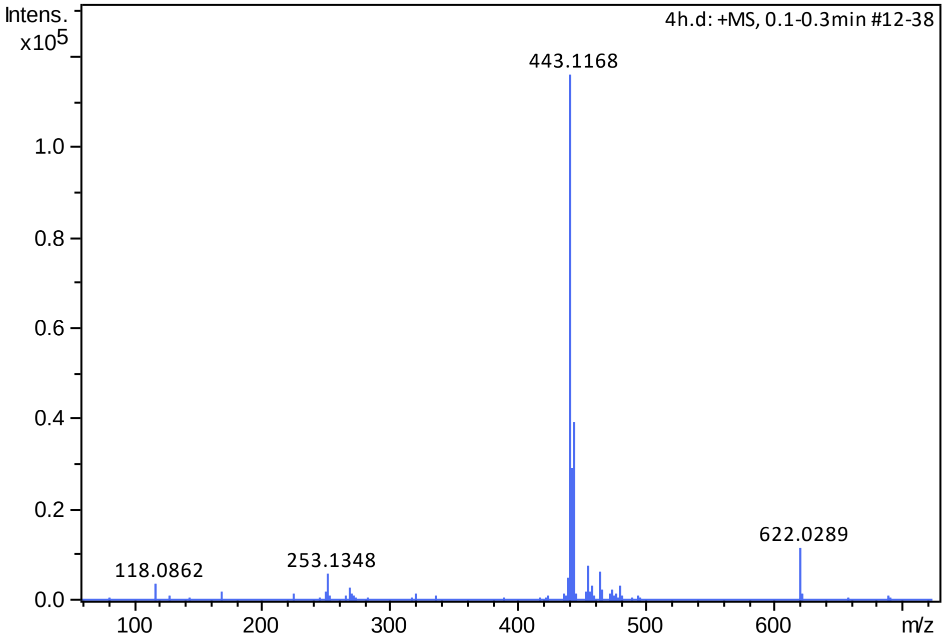

Mass spectra compound 7i

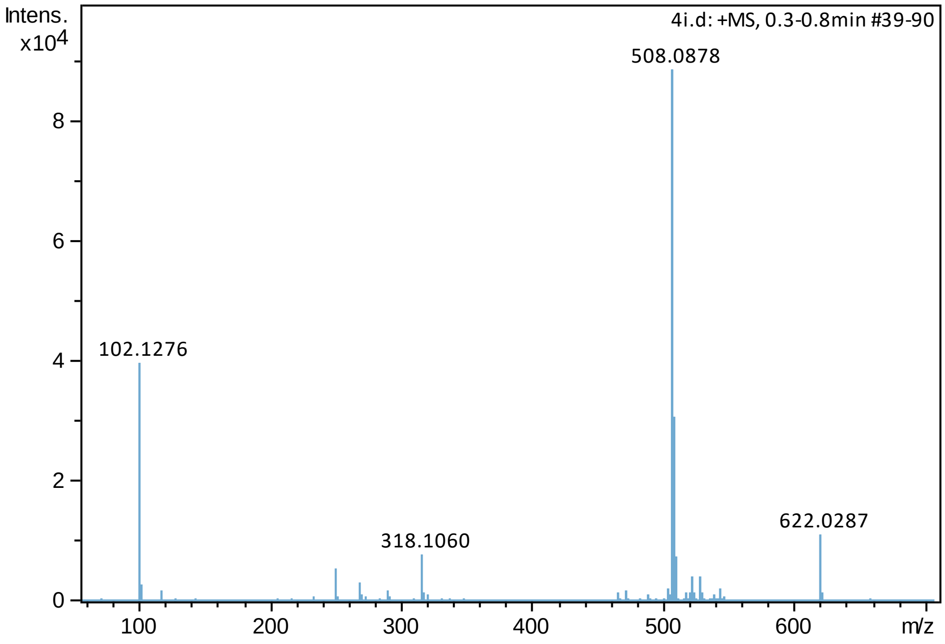

Mass spectra compound 7j

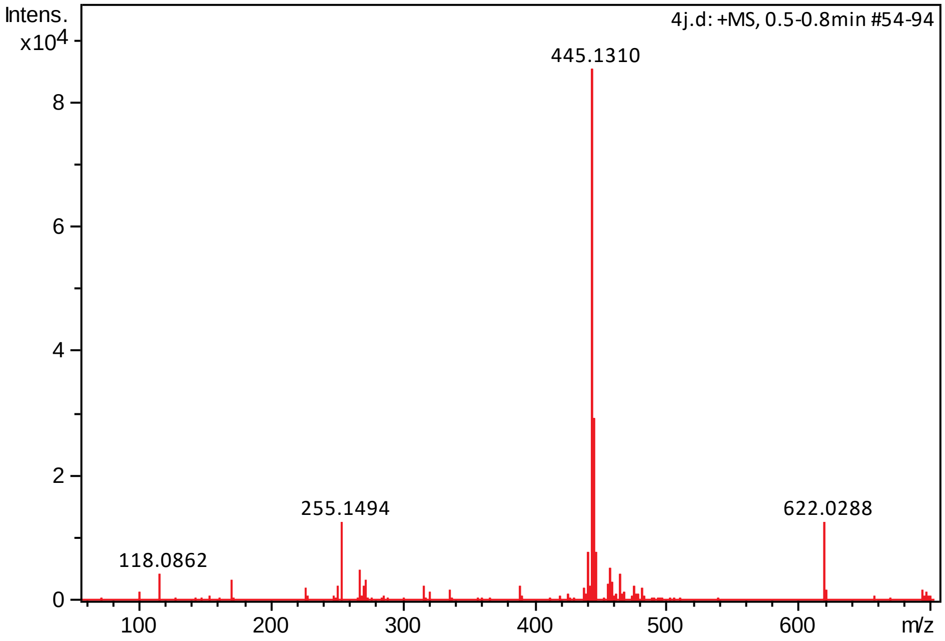

Table S1. Effect of amino naphthoquinone derivatives upon culture growth of *T. cruzi* epimastigote forms expressed as % growth inhibition at 10 μM.

Compound	%GI Epi INC-5	%GI Epi NINOA
**2a**	72±0.01	65±0.07
**2b**	95±0.02	88±0.06
**2c**	100±0.08	100±0.09
**2d**	93±0.01	100±0.5
**2e**	84±0.07	100±0.03
**2f**	95±0.05	100±0.09
**2g**	64±0.0.8	68±0.08
**2h**	100±0.02	100±0.08
**2i**	89±0.03	92±0.04
**4a**	100±0.03	100±0.09
**4b**	98±0.08	96±0.04
**4c**	100±0.09	96±0.03
**4d**	100±0.1	100±0.07
**4e**	96±0.06	86±0.05
**4f**	100±0.07	100±0.07
**4g**	90±0.05	100±0.08
**4h**	87±0.03	98±0.03
**4i**	78±0.03	90±0.07
**4j**	100±0.05	98±0.06
**Bnz**	50±0.36	99±0.02

Table S2. Protein-ligand interaction profiles for naphthoquinones and controls.

TR GR	107ILEB	110LEUB	110SERB	111TYRB	114TYRB	15SERB	18LEUB	33LEUB	19GLUB	34ALAB	22TRPB	37ARGB	335THRB	339THRB	336PROB	340PROB	339ILEB	343ILEB	340ASNB	343ALAB	347ARGB	394SERA	396PHEA	403PHEA	397THRA	404THRA	398PROA	405PROA	399LEUA	406META	402LYSA	409ALAA	457THRA	458ILEA
**2b**	1	4	0	2	7	2	0	5	2	1	0	3	0	0	5	1	6	6	4	0	2	0	1	2	0	2	1	0	5	2	1	0	0	1
**2a**	0	0	0	2	11	0	0	10	1	5	0	6	0	0	7	0	9	9	6	0	6	0	0	0	0	0	0	0	2	1	0	0	0	0
**2c**	0	4	0	1	7	1	0	6	2	2	0	5	0	0	5	1	6	7	6	0	4	0	1	0	0	0	1	0	3	0	0	0	0	1
**2e**	1	2	0	1	4	0	0	1	1	5	1	5	0	0	6	1	5	8	3	0	2	0	8	8	0	0	1	0	2	2	0	0	0	0
**2d**	0	2	0	2	8	0	0	5	2	4	2	6	1	0	6	2	9	10	5	0	0	0	3	4	0	1	0	0	0	2	0	1	0	1
**2f**	1	0	0	2	5	0	1	1	2	3	2	4	0	0	3	1	9	4	2	0	1	0	3	8	0	1	0	0	2	4	0	1	0	0
**2h**	2	1	0	1	5	0	0	2	1	2	0	6	0	0	5	1	5	8	5	0	6	0	1	3	0	1	0	0	4	3	0	0	0	1
**2g**	1	4	0	0	1	0	0	0	0	2	0	2	0	0	3	0	5	0	5	0	0	0	4	3	1	1	0	0	2	4	0	0	0	0
**2i**	1	2	0	0	5	0	0	1	0	2	0	1	0	0	3	1	5	4	4	0	5	0	2	3	0	1	0	0	4	4	0	0	0	0
**7i**	3	4	0	0	2	0	0	0	0	7	0	12	0	0	4	0	7	2	3	0	6	0	4	5	0	0	1	1	7	6	0	1	0	1
**7g**	3	0	0	0	5	0	0	3	1	1	0	6	0	1	6	3	4	9	2	0	4	0	6	3	0	1	0	0	4	3	0	1	0	0
**7a**	1	1	0	2	4	0	0	2	0	1	1	10	0	0	3	2	9	5	2	0	5	0	3	3	0	0	0	0	4	4	0	1	0	0
**7e**	4	0	0	1	5	0	0	2	0	0	0	7	0	0	5	2	8	6	5	2	5	0	8	8	0	1	0	0	8	3	0	3	0	3
**7f**	3	3	0	0	0	0	0	0	0	4	0	8	0	0	1	0	5	0	6	0	6	0	2	3	0	0	0	0	7	7	0	0	0	1
**7h**	1	2	0	2	5	0	0	2	0	1	2	9	0	0	5	1	9	6	6	0	7	0	5	3	1	2	0	0	3	4	0	1	0	0
**7b**	3	2	0	0	2	0	0	0	0	4	0	11	0	0	6	0	11	3	2	0	7	0	4	6	0	0	0	0	11	3	0	1	0	0
**7c**	3	0	0	0	4	0	0	2	0	1	0	5	0	1	4	2	8	7	4	0	4	0	6	5	0	1	0	0	5	2	0	1	0	0
**7d**	3	3	0	0	2	0	0	0	0	3	0	8	0	0	8	0	12	3	2	0	3	0	3	4	0	1	0	0	8	4	0	3	0	1
**7j**	2	6	0	5	4	0	0	0	0	1	2	8	0	0	6	3	8	6	5	0	10	0	7	5	0	0	0	0	4	4	0	1	0	3
**TS2**	2	X	2	8	X	5	0	X	7	X	3	X	0	X	1	X	2	X	0	0	X	1	6	X	1	X	0	X	5	X	1	X	0	0
**NQ-h**	2	2	0	3	7	1	1	5	0	2	2	5	0	0	7	2	9	8	1	0	4	0	3	2	0	1	0	0	3	0	0	1	0	1
**NQ-g**	0	0	0	3	7	1	1	5	0	3	1	6	0	0	7	0	12	7	3	0	2	0	7	6	0	2	0	0	1	2	0	2	0	0
**NQ-d**	5	3	0	1	4	0	1	2	0	2	1	3	0	0	4	1	4	4	2	0	2	0	6	8	0	1	0	0	6	3	0	0	0	2

Table S2. Protein-ligand interaction profiles for naphthoquinones and controls (continued).

TR	GR	459GLYA	465ALAA	461HISA	467HISA	462PROA	468PROA	463THRA	469THRA	466GLUA	472GLUA	467GLUA	473GLUA	469CYSA	470SERA	472ARGA	478ARGA	54VALB	59VALB	59VALB	64VALB
**2b**		5	1	2	3	1	2	0	0	10	4	3	3	0	0	0	0	0	0	0	2
**2a**		5	0	3	2	1	0	0	0	16	5	5	7	0	0	1	2	0	0	0	0
**2c**		3	1	4	3	2	2	0	0	13	6	7	6	0	0	0	0	0	0	0	2
**2e**		1	1	2	3	1	3	0	0	7	3	3	7	0	0	0	3	1	0	0	1
**2d**		1	2	3	3	0	2	0	0	4	3	3	4	0	0	0	4	0	0	0	1
**2f**		2	1	1	1	1	2	0	0	11	2	4	3	0	0	0	2	0	0	0	0
**2h**		3	1	3	2	0	0	0	0	11	0	3	4	0	0	0	1	0	0	2	0
**2g**		3	0	3	4	1	2	0	1	10	0	2	1	0	0	0	4	0	0	0	2
**2i**		1	1	2	1	1	1	0	1	11	0	3	6	0	0	0	0	0	0	0	0
**7i**		2	0	0	2	1	3	2	0	10	0	0	3	0	0	0	3	0	0	0	3
**7g**		3	3	1	4	0	0	0	0	11	1	1	2	0	0	1	7	0	0	1	0
**7a**		3	2	1	2	0	2	0	1	4	1	3	2	0	0	0	3	0	0	0	0
**7e**		2	2	4	2	0	1	0	0	8	0	2	1	0	0	0	7	0	0	1	0
**7f**		1	0	4	5	0	3	1	0	8	0	1	1	0	0	0	0	0	0	0	3
**7h**		3	1	1	1	0	0	0	0	8	1	2	0	1	0	0	4	0	0	0	0
**7b**		0	0	3	2	2	2	1	0	16	0	0	3	0	0	0	1	0	0	0	1
**7c**		3	2	0	4	1	1	0	0	10	1	3	2	0	0	0	9	0	0	0	0
**7d**		1	0	3	3	1	2	2	0	13	0	1	3	0	0	0	5	0	0	0	2
**7j**		3	3	0	5	0	2	0	0	4	0	2	2	0	0	0	4	0	0	1	2
**TS2**		4	X	9	X	0	X	8	X	12	X	1	X	1	2	2	X	0	X	1	X
**NQ-h**		1	2	4	3	3	1	0	0	4	0	0	1	0	0	0	1	1	0	3	1
**NQ-g**		1	0	2	0	2	1	0	0	8	0	0	0	0	0	0	2	0	1	0	0
**NQ-d**		0	1	4	2	4	4	0	0	4	0	1	0	0	0	0	1	2	0	2	1
